# Dlk1 is a novel adrenocortical stem/progenitor cell marker that predicts malignancy in adrenocortical carcinoma

**DOI:** 10.1002/cac2.70012

**Published:** 2025-03-04

**Authors:** Katia Mariniello, James F. H. Pittaway, Barbara Altieri, Kleiton Silva Borges, Irene Hadjidemetriou, Claudio Ribeiro, Gerard Ruiz‐Babot, David S. Tourigny, Jiang A. Lim, Julie Foster, Julie Cleaver, Jane Sosabowski, Nafis Rahman, Milena Doroszko, Constanze Hantel, Sandra Sigala, Andrea Abate, Mariangela Tamburello, Katja Kiseljak‐Vassiliades, Margaret Wierman, Charlotte Hall, Laila Parvanta, Tarek E. Abdel‐Aziz, Teng‐Teng Chung, Aimee Di Marco, Fausto Palazzo, Celso E. Gomez‐Sanchez, David R. Taylor, Oliver Rayner, Cristina L. Ronchi, Carles Gaston‐Massuet, Silviu Sbiera, William M. Drake, Emanuel Rognoni, Matthias Kroiss, David T. Breault, Martin Fassnacht, Leonardo Guasti

**Affiliations:** ^1^ Centre for Endocrinology William Harvey Research Institute Faculty of Medicine and Dentistry Queen Mary University of London London UK; ^2^ Division of Endocrinology and Diabetes Dept. of Medicine University Hospital University of Würzburg Würzburg Germany; ^3^ Division of Endocrinology Boston Children's Hospital Harvard Medical School Boston Massachusetts USA; ^4^ Harvard Stem Cell Institute Cambridge Massachusetts USA; ^5^ Department of Internal Medicine III University Hospital Carl Gustav Carus Technical, University Dresden Dresden Germany; ^6^ School of Mathematics University of Birmingham Birmingham UK; ^7^ Centre for Cancer Biomarkers and Biotherapeutics Barts Cancer Institute Barts and The London School of Medicine and Dentistry Queen Mary University of London, Charterhouse Square London UK; ^8^ Institute of Biomedicine University of Turku Turku Finland; ^9^ Department of Endocrinology Diabetology and Clinical Nutrition University Hospital Zurich (USZ) and University of Zurich (UZH) Zurich Switzerland; ^10^ Section of Pharmacology Department of Molecular and Translational Medicine University of Brescia Brescia Italy; ^11^ Division of Endocrinology Metabolism and Diabetes Department of Medicine University of Colorado School of Medicine Aurora Colorado USA; ^12^ Division of Endocrinology Metabolism and Diabetes at Rocky Mountain Regional Veterans Affair Medical Center Washington District of Columbia USA; ^13^ Department of Surgery St Bartholomew's Hospital, West Smithfield London UK; ^14^ Department of Surgery University College London Hospitals NHS Foundation Trust London UK; ^15^ Department of Endocrinology University College London Hospitals NHS Foundation Trust London UK; ^16^ Department of Endocrine and Thyroid Surgery Hammersmith Hospital, Imperial College London London UK; ^17^ Endocrine Section G.V. (Sonny) Montgomery VA Medical Center and the Department of Pharmacology and Toxicology University of Mississippi Medical Center Jackson Mississippi USA; ^18^ Department of Clinical Biochemistry (Synnovis Analytics) King's College Hospital London UK; ^19^ Institute of Metabolism and System Research College of Medical and Dental Sciences University of Birmingham Birmingham UK; ^20^ Centre for Cell Biology & Cutaneous Research Blizard Institute Barts and The London School of Medicine and Dentistry Queen Mary University of London London UK; ^21^ Department of Internal Medicine IV LMU University Hospital, LMU Munich München Germany

**List of Abbreviations**
ACC: Adrenocortical carcinoma
DLK1: Delta‐like non‐canonical Notch ligand 1
ENS@T: European Network for the Study of Adrenal Tumors
GDX: Gonadectomy
PDGFRα: Platelet‐derived growth factor receptor alpha

1

Adrenocortical carcinoma (ACC) is a rare malignancy with no widely available biomarkers and commonly presents at later stages with a bleak prognosis [1]. Dysregulation of signaling pathways involved in the organogenesis and homeostasis of the adrenal cortex is implicated in its pathogenesis [2]. The paternally expressed, cleavable protein delta‐like non‐canonical Notch ligand 1 (DLK1) is expressed in rat adrenocortical progenitor cells [3] and in clusters of relatively undifferentiated cells in the human adrenal gland [4]. Its expression is rare in most adult human tissues but has been reported across various cancers, often associated with worse survival [5]. Here we define the role of DLK1 in adrenocortical development, self‐renewal, and the development and progression of ACC.

Dlk1^+^ cells were present in both the capsule and cortex during embryonic development but became restricted to the capsule postnatally in both male and female mice (Supplementary Figure S1), with minimal overlap in expression with Axin‐2 (Wnt‐active) cells, their early descendants, and platelet‐derived growth factor receptor alpha (PDGFRα), a marker of mesenchymal stem/fibroblastic cells (Supplementary Figure S2). Dlk1 cells were rarely positive for Ki‐67, whereas *Gli1* expression in the capsule, unlike Dlk1, remained high during development and throughout postnatal life (Supplementary Figure S3). Genetic lineage tracing using inducible *Dlk1^CreERT2/+^*; *Rosa^tdTomato/+^* mice showed that Dlk1^+^ cells functioned as adrenocortical stem cells during development (Figure 1A‐F), but were largely dormant postnatally and inactive during postnatal adrenocortical remodeling (Supplementary Figure S4).

**FIGURE 1:**
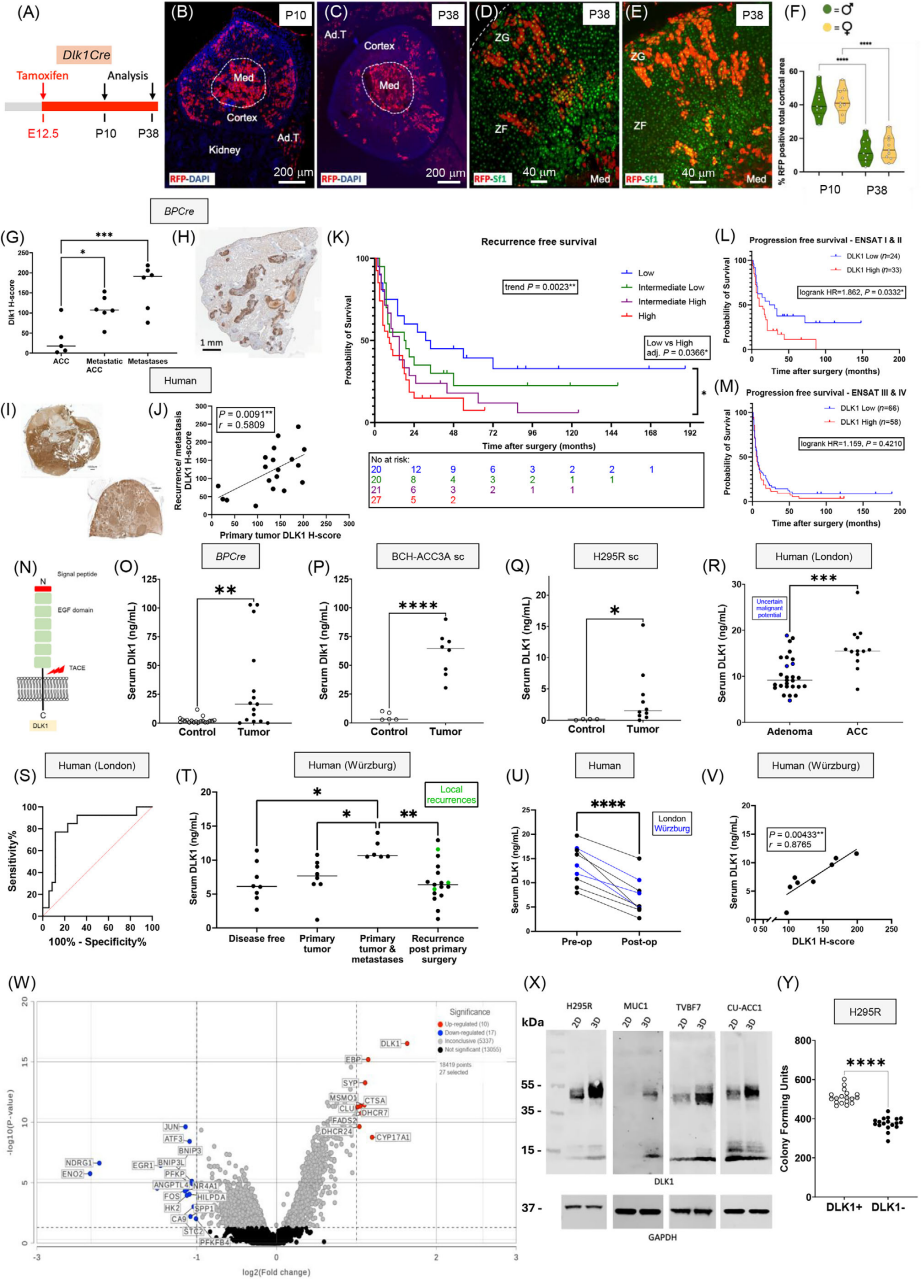
**Dlk1 is an adrenocortical stem/progenitor cell marker that predicts malignancy in adrenocortical carcinoma in mice and humans**. (A) Schematic of *Dlk1^CreERT2/+^*; *Rosa^tdTomato/+^* mice (*Dlk1Cre*) injected with tamoxifen for fate mapping experiments. Dlk1^+^ cells and their progeny were labelled with tdTomato (visualized with an anti‐Red Fluorescence Protein [RFP] antibody) upon tamoxifen injection. (B‐E) When dams were injected with tamoxifen at E12.5, and adrenals were analyzed at both P10 and P38, clusters and columns of RFP^+^/Sf1^+^ cells (representing Dlk1 progeny) spanned the entire width of the cortex in both males (D) and females (E). As expected, RFP was also detected in the medulla. (F) Dlk1 progeny were significantly decreased at P38 compared to P10, with females showing a small, non‐significant trend toward more Dlk1 progeny than males. (G) In *BPCre* mice, Dlk1 expression increased stepwise from non‐metastatic primary ACC to metastatic primary ACC and then to metastatic lesions. (H) Intense DLK1 expression was observed in lung metastases. (I) In humans, DLK1 expression was consistent in primary (upper left) and recurrent (lower right) tumors in the same patient. 19 secondary disease specimens were available from patients whose primary tumors were included in the study. (J) DLK1 expression level in secondary tumors positively correlated with those in primary tumors. (K) Categorizing DLK1 expression levels into quartiles (based on median and interquartile range values), higher DLK1 levels were associated with stepwise increase in the risk of disease recurrence (median RFS: low DLK1 = 32.5 months, low‐intermediate DLK1 = 18.5 months, high‐intermediate DLK1 = 15 months, and high DLK1 = 9 months). This was significant by the log‐rank test for trend across the four groups (χ^2^ = 9.263) and when comparing high versus low DLK1 expression groups directly. (L) Higher DLK1 expression was associated with an increased risk of disease progression in ENSAT stage I & II disease (*n* = 57, median PFS: high DLK1 = 10 months versus low DLK1 = 27.5 months, HR 1.863, 95% CI = 1.038‐3.340). (M) In ENSAT stage III & IV groups, median PFS was comparable (*n* = 121, high DLK1 = 6 months versus low DLK1 = 7 months, HR = 1.159, 95% CI = 0.793‐1.694). There was no significant difference in mean Ki‐67% between ENSAT stage I & II group (20.24 ± 16.68) and stage III & IV group (19.57 ± 16.28) in this cohort. (N) Illustration of DLK1 structure, highlighting the ectodomain cleaved by TACE. (O‐Q) Serum Dlk1 levels were significantly higher in *BPCre* mice than in age‐matched controls. This was also observed in subcutaneous tumor mouse models injected with BPCre tumor‐derived BCH‐ACC3A cells (P) and H295R human ACC cells (Q). (R) In humans, pre‐operative serum DLK1 levels in the London prospective discovery cohort were significantly higher in ACC (16.81 ± 4.876ng/mL) than in benign adrenocortical adenomas (10.54 ± 4.417ng/mL). (S) Receiver operating characteristic (ROC) curve using all pre‐operative values demonstrated that serum DLK1 predicted ACC diagnosis with high accuracy (AUC 0.824 ± 0.072, *P* < 0.001). (T) In the Würzburg validation cohort, serum DLK1 levels were higher in patients with ENSAT stage IV disease than in those with recurrent disease following primary surgery (11.46 ± 1.459ng/mL versus 6.749 ± 3.016ng/mL), disease‐free patients (6.666 ± 2.855ng/mL), and patients with isolated primary tumors (11.46 ± 1.459ng/mL versus 7.357 ± 2.913ng/mL). (U) Following primary ACC resection, serum DLK1 levels significantly decreased compared to pre‐operative levels (mean decrease = 6.568 ± 2.565ng/mL). (V) Pre‐operative serum DLK1 levels significantly correlated with primary ACC DLK1 H‐score in the same patients. (W) Volcano plot of differentially expressed genes in DLK1^+^ versus DLK1^−^ tumor areas using spatial transcriptomics. Applying a fold‐change cutoff of > 2 or < 2, 10 genes were significantly upregulated, and 17 genes were significantly downregulated. Among the 9 upregulated genes (excluding *DLK1*), 5 were involved in cholesterol synthesis (*EBP*, *DHCR7*, *DHCR24*, *MSMO1*) and fatty acids metabolism (*FADS2*). Other upregulated genes included those involved in steroidogenesis (*CYP17A1*), vesicular and cholesterol binding (*SYP*), and cathepsin (*CTSA*) and clusterin (*CLU*). Downregulated genes included pro‐apoptotic genes (*BNIP3*, *BNIP3L*, *NR4A1*) and transcriptional regulators of differentiation (*EGR1*, *FOS*, *JUN*). (X) Western blot analysis showing increased DLK1 expression across the indicated ACC cell lines when cultured in 3D spheroid compared to 2D culture. (Y) H295R cells were fluorescence‐activated cell sorted (FACS) into DLK1^+^ and DLK1^−^ populations. DLK1^+^ cells generated significantly more colony‐forming units than DLK1^−^ cells after 21 days in culture. Data are displayed as individual points, with horizontal bars representing the mean. *P < 0.05, **P < 0.01, ***P < 0.001, ****P < 0.0001. Abbreviations: E, embryonic day; EGF, epidermal‐like growth factor; Med, Medulla; P, postnatal day; PFS, progression‐free survival; RFS, recurrence‐free survival; sc, subcutaneous; TACE, TNFα converting enzyme.

Capsular‐like cells are pathognomonic of subcapsular hyperplasia (SH), a histological hallmark in mouse adrenals that occurs spontaneously in aged females and in certain strains/transgenic models after gonadectomy (GDX) [6]. SH foci are thought to represent a morphological continuum progressing toward adrenocortical tumors. Dlk1 was not expressed in SH or in subsequent tumors in two GDX mouse models (Supplementary Figure S5). Moreover, spontaneous SH foci in aged mice were neither enriched in nor derived from Dlk1‐expressing cells (Supplementary Figure S6), supporting the hypothesis that SH results from a de‐differentiation event [7]. Interestingly, Dlk1 was re‐expressed in an autochthonous mouse model of ACC, in which concomitant inactivation of *Trp53* and activation of *Ctnnb1*, driven by the aldosterone synthase promoter (*BPCre*) [8], leads to ACC formation with high penetrance. In 23 tumor samples from 17 mice (9 female), Dlk1 expression was low or absent in benign and pre‐malignant tumors, moderate in localized ACC, and higher in metastatic disease, both in the primary tumors and in lung metastases. There was a stepwise increase of Dlk1 expression with disease severity, and a positive correlation between Dlk1 expression and age (Figure 1G‐H, Supplementary Figure S7). These results indicate that in the *BPCre* model, Dlk1, rather than marking the cell of origin, is re‐expressed in ACC, potentially conferring cancer stem cell characteristics.

In a prospective discovery cohort of 73 consecutive patients (26 male) undergoing adrenalectomy in London, UK (Supplementary Table S1), DLK1 expression was significantly higher in ACC than in benign adrenal disease and normal adrenals (Supplementary Figure S8A). This finding was validated in a larger cohort from Würzburg, Germany, comprising 178 ACC tumor samples from 159 patients (53 male) (Supplementary Table S2). DLK1 expression was ubiquitous and heterogenous, with apparent clones of DLK1‐positive cells, similar to those observed in *BPCre* mice. DLK1 expression was not correlated with age, sex, or tumor size and remained constant across different European Network for the Study of Adrenal Tumors (ENS@T) tumor stages, hormonal activity of tumors, Weiss score, and Ki‐67% (Supplementary Figure S8B‐H). As in *BPCre* mice, DLK1 expression was present in recurrent human disease and could clearly identify metastases from background tissue. There was a significant positive correlation between DLK1 expression in primary tumors and in recurrent/metastatic disease in the same patients (Figure 1I‐J), marking DLK1 expression as a disease defining feature of disease progression.

In primary ACC (*n* = 88), higher DLK1 expression was associated with a stepwise increase in the risk of disease recurrence, which remained independently significant in multivariate Cox regression analysis (Figure 1K, Supplementary Table S3, Supplementary Figure S8I‐J). In all ACC samples (*n* = 176), higher DLK1 expression trended toward an increased risk of disease progression, though this did not reach statistical significance in multivariate Cox analysis (*P* = 0.079) (Supplementary Figure S8K, Supplementary Table S3). However, higher DLK1 expression was significantly associated with an increased risk of progression in ENS@T stage I & II disease (Figure 1L‐M). These data suggest the metastatic potential of ACC may be influenced by DLK1 levels. RNA sequencing of the ACC cell line H295R, with DLK1 overexpression and knockdown, revealed that higher DLK1 expression was associated with lower expression of immune signaling gene set, suggesting that the carcinogenic role of DLK1 may, in part, be mediated through mechanisms associated with senescence‐induced immune remodeling [9] (Supplementary Figure S9A‐E).

DLK1 has a cleavable ectodomain that is detectable in serum. Serum Dlk1 levels were significantly higher in *BPCre* mice (compared to age‐matched controls) and in two subcutaneous tumor mouse models: one using the *BPCre* tumor‐derived cell line BCH‐ACC3A [10] and another injected with H295R cells (Figure 1N‐Q). In all cases, there was a strong positive correlation between tumor size and serum DLK1 levels (Supplementary Figure S10). In humans, pre‐operative serum DLK1 levels were significantly higher in ACC than in benign adrenocortical adenomas in the London cohort and could predict the diagnosis of ACC with high sensitivity and specificity (Figure 1R‐S). This finding was validated in the German cohort, where significantly higher serum DLK1 levels were observed in patients with a greater disease burden (Figure 1T, Supplementary Table S4). As in tissue, serum DLK1 levels did not correlate with other prognostic or clinicopathological features (Supplementary Figure S11A‐F). Post‐operative blood samples showed a significant reduction in DLK1 levels after tumor resection (Figure 1U). Pre‐operative serum DLK1 levels positively correlated with tissue DLK1 expression in both cohorts (Figure 1V, Supplementary Figure S11G). These findings indicate that serum DLK1 is derived from ACC, with levels reflecting the DLK1 expression of the primary tumor and the extent of disease.

Spatial whole‐transcriptome profiling was performed on DLK1^+^ and DLK1^−^ regions within four human ACCs. Surprisingly, steroid biosynthesis was the gene ontology pathway most enriched in the DLK1^+^ group, consistent with the upregulation of cholesterol synthesis genes, suggesting that DLK1^+^ areas have higher steroidogenic potential than DLK1^−^ areas (Figure 1W, Supplementary Figures S12‐S13). This finding was further supported by increased expression of adrenal differentiation genes with higher DLK1 dosage in the H295R transcriptomic data (Supplementary Figure S9F‐H). To further investigate this apparent paradox of enhanced steroidogenic potential in ACC cells expressing an adrenocortical stem cell marker, four different human ACC cell lines (H295R, MUC‐1, TVBF7 and CU‐ACC1) and one mouse ACC cell line (BCH‐ACC3A) were cultured as spheroids. DLK1 expression was significantly enhanced in 3D versus 2D culture in H295R, TVBF7, and CU‐ACC1, and interestingly, *de novo* expression of DLK1 protein was observed in MUC‐1 (Figure 1X, Supplementary Figure S14A‐F). Liquid chromatography with tandem mass spectrometry revealed that 3D spheroids had significantly increased output of steroids compared to 2D cells in H295R, CU‐ACC1, and BCH‐ACC3A, with a trend toward increased steroidogenesis in MUC‐1 and TVBF7 (Supplementary Table S5). Fluorescence‐activated cell sorting showed that DLK1^+^ cells generated significantly more colony‐forming units than DLK1^−^ populations after 21 days in culture (Figure 1Y, Supplementary Figure S14G‐H). These findings suggest that ACC cells expressing a bona fide adrenocortical stem cell marker possess superior steroidogenic potential while retaining some progenitor cell features, providing a possible explanation for the negative prognostic impact of DLK1 expression in ACC.

These data define Dlk1 as a novel adrenocortical stem/progenitor cell marker with a role in both adrenocortical organogenesis and malignancy development. Expression data from mice and human ACC indicate that DLK1 is associated with increased malignancy and tumor aggressiveness. Furthermore, DLK1 holds promise as a biomarker for the diagnosis, prognosis, and follow‐up of patients with ACC, particularly through serum measurements using a benchtop assay. Further larger prospective studies are needed to confirm this role, along with investigations into DLK1 as a potential therapeutic target in ACC, given its preferential expression in this malignancy.

## AUTHOR CONTRIBUTIONS

*Conceptualization*: Leonardo Guasti, James F.H. Pittaway, and Katia Mariniello. *Methodology*: Leonardo Guasti, James F.H. Pittaway, Katia Mariniello, Barbara Altieri, Irene Hadjidemetriou, Silviu Sbiera, Matthias Kroiss, Martin Fassnacht, William M. Drake, Kleiton Silva Borges, and David T. Breault. *Validation*: Kleiton Silva Borges, Claudio Ribeiro, Katia Mariniello, James F.H. Pittaway, Barabara Altieri, Jiang A. Lim, David T. Breault, David S. Tourigny and Charlotte Hall. *Formal analysis*: James F.H. Pittaway, Katia Mariniello, Barbara Altieri, and Kleiton Silva Borges. *Investigation*: Gerard Ruiz‐Babot, Oliver Rayner, David R. Taylor, James F.H. Pittaway, Katia Mariniello, Barbara Altieri, Leonardo Guasti, Silviu Sbiera, Carles Gaston‐Massuet, and Emanuel Rognoni. *Resources*: Sandra Sigala, Andrea Abate, Mariangela Tamburello, Katja Kiseljak‐Vassiliades, Margaret Wierman, Laila Parvanta, Tarek E. Abdel‐Aziz, Teng‐Teng Chung, Aimee Di Marco, Fausto Palazzo, Celso E. Gomez‐Sanchez, Constanze Hantel, Julie Foster, Julie Cleaver, Jane Sosabowski, Nafis Rahman, Milena Doroszko, and Cristina L. Ronchi. *Data curation*: James F.H. Pittaway, Katia Mariniello, and Leonardo Guasti. *Writing‐original draft*: James F.H. Pittaway, Katia Mariniello, and Leonardo Guasti. *Writing‐review and editing*: all authors. *Supervision*: Leonardo Guasti, William M. Drake, Martin Fassnacht, Matthias Kroiss, David T. Breault. *Project administration*: Leonardo Guasti.

## CONFLICT OF INTEREST STATEMENT

The authors declare no potential conflicts of interest regarding the research, authorship, and/or publication of this article.

## FUNDING INFORMATION

This work was supported by the MRC (MR/X021017/1, MR/S022155/1), BBSRC (BB/V007246/1), Barts Charity (MGU0436), Rosetrees Trust (M355‐F1), The Medical College of Saint Bartholomew's Hospital Trust, the German Research Foundation (Deutsche Forschungsgemeinschaft, 314061271), and the National Institutes of Health Physician‐Scientist Career Development Award (R01DK123694).

## ETHICS APPROVAL AND CONSENT TO PARTICIPATE

Human adrenal specimens were collected from patients undergoing surgery at St Bartholomew's, University College and Hammersmith Hospitals, London, after obtaining written informed consent from participants and in accordance with the study protocol Genetics of endocrine tumors (REC: 06/Q0104/133). In Germany, all tissue was collected under the ENS@T research ethical agreement (No. 88/11) at the Universitätsklinikum Würzburg. All patients provided informed consent. All clinical data were collected through the ENS@T database (registry.ensat.org).

## Supporting information



Supporting Information

## Data Availability

In all graphs, data are presented as individual values for transparency. Spatial transcriptomic and cell line transcriptomic data have been deposited in the National Center for Biotechnology Information (NCBI) Gene Expression Omnibus (GEO) under accession numbers GSE277486 and GSE286393, respectively.
