# Supplementary material for: Dlk1 is a novel adrenocortical stem/progenitor cell marker that predicts malignancy in adrenocortical carcinoma
Source: Cancer Commun (Lond). 2025 Mar 4;45(6):663–8. doi: 10.1002/cac2.70012 (PMC12187577; doi:10.1002/cac2.70012)
Supplement: Supplementary file 1 — Supporting Information [file CAC2-45-663-s001.docx]

**Supplementary Materials**

**Dlk1 is a novel adrenocortical stem/progenitor cell marker that predicts malignancy in adrenocortical carcinoma**

Katia Mariniello^1,†^, James F.H. Pittaway^1,†,*^, Barbara Altieri^2^, Kleiton Silva Borges^3,4^, Irene Hadjidemetriou^1^, Claudio Ribeiro^3,4^, Gerard Ruiz-Babot^3,5^, David S. Tourigny^6^, Jiang A. Lim^1^, Julie Foster^7^, Julie Cleaver^7^, Jane Sosabowski^7^, Nafis Rahman^8^, Milena Doroszko^8^, Constanze Hantel^9^, Sandra Sigala^10^, Andrea Abate^10^, Mariangela Tamburello^10^, Katja Kiseljak-Vassiliades^11,12^, Margaret Wierman^11,12^, Charlotte Hall^1^, Laila Parvanta^13^, Tarek E. Abdel-Aziz^14^, Teng-Teng Chung^15^, Aimee Di Marco^16^, Fausto Palazzo^16^, Celso E. Gomez-Sanchez^17^, David R. Taylor^18^, Oliver Rayner^18^, Cristina L. Ronchi^19^, Carles Gaston-Massuet^1^, Silviu Sbiera^2^, William M. Drake^1^, Emanuel Rognoni^20^, Matthias Kroiss^2,21^, David T. Breault^3,4^, Martin Fassnacht^2^, Leonardo Guasti^1^

^1^Centre for Endocrinology, William Harvey Research Institute, Faculty of Medicine and Dentistry, Queen Mary University of London, London, UK.

^2^Division of Endocrinology and Diabetes, Dept. of Medicine, University Hospital, University of Würzburg, Würzburg, Germany.

^3^Division of Endocrinology, Boston Children’s Hospital, Harvard Medical School, Boston, Massachusetts, USA.

^4^Harvard Stem Cell Institute, Cambridge, Massachusetts, USA.

^5^Department of Internal Medicine III, University Hospital Carl Gustav Carus, Technical, University Dresden, Dresden, Germany.

^6^School of Mathematics, University of Birmingham, Birmingham, UK.

^7^Centre for Cancer Biomarkers and Biotherapeutics, Barts Cancer Institute, Barts and The London School of Medicine and Dentistry, Queen Mary University of London, Charterhouse Square, London, UK.

^8^Institute of Biomedicine, University of Turku, Turku, Finland.

^9^Department of Endocrinology, Diabetology and Clinical Nutrition, University Hospital Zurich (USZ) and University of Zurich (UZH), Zurich, Switzerland.

^10^Section of Pharmacology, Department of Molecular and Translational Medicine, University of Brescia, Brescia, Italy.

^11^Division of Endocrinology, Metabolism and Diabetes, Department of Medicine, University of Colorado School of Medicine, Aurora, Colorado, USA.

^12^Division of Endocrinology, Metabolism and Diabetes at Rocky Mountain Regional Veterans Affair Medical Center, Washington, DC, USA.

^13^Department of Surgery, St Bartholomew’s Hospital, West Smithfield, London, UK.

^14^Department of Surgery, University College London Hospitals NHS Foundation Trust, London, UK.

^15^Department of Endocrinology, University College London Hospitals NHS Foundation Trust, London, UK.

^16^Department of Endocrine and Thyroid Surgery, Hammersmith Hospital, Imperial College London, London, UK.

^17^Endocrine Section, G.V. (Sonny) Montgomery VA Medical Center and the Department of Pharmacology and Toxicology, University of Mississippi Medical Center, Jackson, Mississippi, USA.

^18^Department of Clinical Biochemistry (Synnovis Analytics), King’s College Hospital, London, UK.

^19^Institute of Metabolism and System Research College of Medical and Dental Sciences, University of Birmingham, Birmingham, UK.

^20^Centre for Cell Biology & Cutaneous Research, Blizard Institute, Barts and The London School of Medicine and Dentistry, Queen Mary University of London, London, UK.

^21^Department of Internal Medicine IV, LMU University Hospital, LMU Munich, München, Germany.

^†^Katia Mariniello and James F.H. Pittaway contributed equally for this work (co-first authors).

^*^Corresponding author:

James F. H. Pittaway; Centre for Endocrinology, William Harvey Research Institute, Barts and the London School of Medicine and Dentistry, Queen Mary University of London, London, EC1M 6BQ, UK; Email: [j.pittaway@qmul.ac.uk](mailto:j.pittaway@qmul.ac.uk); Telephone: +44 (0)20 7882 5555.

**Supplementary Material and Methods**

**Genetic lineage tracing**

Mice were housed in a 12-hour light/dark cycle in a temperature- and humidity-controlled room (21 °C, 55% humidity) with constant access to food and water. Experimental procedures in the UK were under the terms of a UK government Home Office license (PPL P48019841). All mice were maintained on a C57BL/6 background and included Rosa26^CAGLoxpSTOPLoxpTdTomato^ (RRID:IMSR_JAX:007914), Dlk1CreERT2 (a gift from Prof. Fiona Watt, Kings College, London, UK), PDGFRα-H2BEGFP (RRID:IMSR_JAX:007669**)**. Rosa26^CAGLoxpSTOPLoxpTdTomato^ mice were crossed with Dlk1CreERT2 mice to generate DLK1-CreER; Rosa26C^TdTomato/+^ mice. Axin2Cre:ERT2/+ mice and Rosa^YFP/YFP^ mice were purchased from Jackson laboratories. These mice were crossed to produce Axin2CreERT2/+; Rosa^YFP/YFP^ mice for lineage tracing studies. Tamoxifen (200 mg/g in corn oil, administered via intraperitoneal injection or oral gavage) was given to dams or postnatal mice, with chase times varying as described in the main text. Initial experiments aimed to determine a tamoxifen dose resulting in >80% recombination after 6 days, assessed by immunohistochemistry (IHC) on consecutive sections with anti-RFP and anti-Dlk1 antibodies. No leakage was observed in randomly selected adrenals from sham-injected Dlk1CreERT2;Rosa26C^TdTomato/+^ mice stained with anti-RFP.

For Zona Fasciculata (ZF) remodeling, two inductions regiments were used: (1) *Dlk1Cre* mice at P60 and P460 were treated with tamoxifen (200 mg/g in corn oil, oral gavage) on day 0 and 3, followed by dexamethasone (6.5 μg/g in corn oil, oral gavage) or vehicle on days 1, 2, 4 and 5. (2) *Dlk1Cre* mice at P60 and P460 were treated with tamoxifen on day 0, 3 and 6, and with dexamethasone or vehicle on days 1, 2, 4 and 5. Corticosterone levels were measured using an ELISA (Enzo Life Sciences).

For Zona Glomerulosa (ZG) remodeling, *Dlk1Cre* mice at P50 and P70 were assigned to experimental groups according to different sodium chloride contents in their chow (standard diet, low sodium 0.003%, high sodium 3.3%, SAFE^®^ Complete Care Competence) for 8 days. Tamoxifen (200 mg/g in corn oil, oral gavage) was administered on days 0 and 3.

Adult mice underwent transcardiac perfusion with phosphate-buffered saline (PBS), followed by fixation with 4% paraformaldehyde (PFA) in PBS. Embryonic and postnatal adrenals were fixed/post-fixed in PFA before paraffin embedding.

Timed pregnancies were achieved by overnight mating females and males. The presence of a vaginal plug the following morning was considered as embryonic day (E) 0.5.

**Murine ACC models**

The protocols for animal experiments were approved by Boston Children’s Hospital’s Institutional Animal Care and Use Committee. The *BPCre* mouse model of spontaneous ACC was bred as described previously [1]. These mice express activated *Ctnnb1* (β-catenin) and mutated *Trp53* (p53) (AS^Cre/+^::Trp53^flox/flox^::Ctnnb^flox(ex3)/+^) in the adrenal, leading to spontaneous metastatic ACC. The derivation of the BCH-ACC3A cell line from a *BPCre* tumor is described elsewhere [2]. Tumors were weighed before fixation in PFA and paraffin embedding. Following retro-orbital blood collection, serum was stored at -80°C until analysis. Samples were thawed and analyzed using the Mouse Dlk1 ELISA Kit (Invitrogen EM66RB) following the manufacturer’s instructions.

The study on H295R subcutaneous injection in mice was performed in compliance with Home Office PPL PP6127261. H295R cells in the exponential growth phase were collected, and cell suspensions (10 × 10^6^ cells/100 μL in 10% Tween-80 PBS) were inoculated subcutaneously into the right flanks of 9-week-old female NMRI-Foxn1nu/nu mice (Janvier labs). The tumor take rate was 80%. Tumor volume (mm^3^) was assessed via caliper measurement twice a week and calculated using the formula: length x width^2^/2. Pentobarbital anesthetized mice were exsanguinated by cardiac puncture when tumors reached different sizes, blood was collected for serum human DLK1 measurements (AdipoGen life Sciences). Tumors were also measured after collection before fixation in PFA, paraffin embedding, and processing for DLK1 IHC.

**Gonadectomized mice model**

All procedures in the current experiments were approved by the University of Turku Ethical Committee on Use and Care of Animals. The maintenance of DBA/2J mice and inhibin α subunit promoter (Inhα)/Simian virus 40 T-antigen mice, and the gonadectomy procedures, have been described previously [3, 4].

**Ethics approval and consent to participate**

Human adrenal specimens were collected from patients undergoing surgery at St Bartholomew’s, University College and Hammersmith Hospitals, London, after obtaining written informed consent from participants. This study was conducted under the protocol *Genetics of endocrine tumors* (REC: 06/Q0104/133).

In Germany, all tissue was collected under the ENS@T research ethical agreement (No. 88/11) at the Universitätsklinikum Würzburg. All patients provided informed consent. All clinical data were collected through the ENS@T database (registry.ensat.org).

**Human tissue processing**

Samples were fixed in 4% paraformaldehyde (PFA) for 10-24 hours at 4^o^C and embedded in paraffin. Sections were cut at 2-8μm using a rotary microtome (Thermo scientific) and transferred onto SuperFrost Plus Adhesion slides (VWR).

**Immunohistochemistry and section analysis**

Formalin-fixed, paraffin-embedded (FFPE) sections were deparaffinized in xylene (three 10-minute washes), washed in 100% ethanol (two 10-minute washes), and incubated in a 3% hydrogen peroxide solution in methanol for 30 minutes at room temperature (RT) to block endogenous peroxidase activity. After dehydration in a descending ethanol series (100%, 90%, 70%, and 50%, each for 10 minutes), sections were washed in ddH_2_O, submerged in citrate buffer (Vector) for 20 minutes at 95^o^C, and then allowed to gradually reach RT. Sections were then blocked with 10% goat serum in PBS-Triton 0.1% (T-PBS) containing 4 drops/ml of Avidin solution (Avidin/Biotin Blocking Kit, Vector Labs SP-2001) for 1 hour and then incubated overnight with the primary antibody (Supplementary Table S6) containing 4 drops/ml of Biotin Solution (Avidin/Biotin Blocking Kit, Vector Labs) at RT. Slides were washed with T-PBS and incubated with a biotinylated goat anti-rabbit secondary antibody (Supplementary Table S7) diluted in T-PBS for 2 hours at RT. After further washes in T-PBS, slides were incubated with the Avidin-Biotin Complex (Vector Labs, PK-6100) at RT for 1 hour. Following washes, sections were developed with 3,3’-diaminobenzidine (Vector Labs) and counter-stained with Gill hematoxylin (Sigma). Slides were dehydrated, incubated with xylene and mounted using Vectamount mounting medium (Vector Labs).

In Germany, IHC was performed on full sections of each tumor sample. Slides were deparaffinized in xylene (two 10-minute washes) and rehydrated in ethanol (100%, 90%, 80%, and 70%, each for 5 minutes). After 5 washes in ddH^2^O, high temperature antigen retrieval was performed using a 10 mM citric acid monohydrate buffer (pH 6.5, Sigma) in a pressure cooker (Silit) for 13 minutes. After cooling at RT for 20 minutes, slides were washed five times in ddH2O, and endogenous peroxidase activity was blocked in the dark with 3% hydrogen peroxide solution in methanol for 10 minutes. After five washes in ddH_2_O, non-specific protein-antibody interactions was blocked with 20% human AB serum (Sigma) in PBS for one hour at RT in the dark. Primary antibody was then added in PBS, and slides were incubated at RT for one hour. After five washes in PBS, signal amplification was achieved using the HiDef DetectionTM HRP Polymer System for 20 minutes at RT, following the manufacturer’s instructions. After three 2-minute washes in PBS, slides were then developed for 10 min using the DAB Substrate Kit (Vector Labs) according to the manufacturer’s instructions. Development was stopped with three washes in tap water. Nuclei were counterstained with Mayer’s hematoxylin for 3 minutes. Slides were then washed for 2 minutes in running tap water, and sequentially dehydrated for 2 minutes in 70%, followed by two changes of 100% ethanol and xylene. Finally, slides were mounted with Entellan (Merck).

In London, slides were scanned at 20x magnification with a Grundium Ocus slide scanner (Grundium). Scanned images were imported into QuPath (Open-source software for digital pathology image analysis) [5], and manually annotated. Positive cell detection software was used to generate a H score (maximum of 300) for each section.

In Germany, slides were scanned at 20× magnification on an Aperio Versa microscope (Leica Biosystems, Germany). Images were checked, manually annotated, and then analyzed using the Aperio Positive Pixel Count software (Leica). Staining intensity and distribution were calculated by the software, and a H score (maximum of 300) was generated for each sample.

**Immunofluorescence**

For immunofluorescence, the IHC protocol was followed, omitting the hydrogen peroxide step. Sections were incubated with primary antibodies (Supplementary Table S6) overnight at RT, washed in T-PBS, and incubated with fluorescently labelled secondary antibodies (Supplementary Table S8). Sections were then counterstained with 4’,6-diamidino-2-phenylindole (DAPI, Sigma) before mounting. Images were acquired using a Leica DM5500B microscope, equipped with a DCF365FX camera (Leica), and then processed with Abode Photoshop CS6.

**Cell culture maintenance**

NCI-H295R cells were maintained in DMEM/F-12 HAM (1:1)/GlutaMAX (Gibco), supplemented with 1% Insulin-Transferrin-Selenium (Scientific lab) and 2.5% NuSerum (Scientific lab). CU-ACC1 cells were cultured in F12 Nutrient ham (Gibco) and DMEM-high glucose, pyruvate (Gibco) at a 3:1 V/V ratio, supplemented with 10% FBS, 0.4ug/ml Hydrocortisone, 5ug/ml Insulin, 8.4ng/ml Cholera toxin, 24ug/ml Adenine, and 10ng/ml EGF. TVBF7 and MUC1 cells were maintained in DMEM/F-12 HAM (1:1) + GlutaMAX (Gibco) with 10% FBS.

All cell lines were supplemented with 1% Penicillin-streptomycin and cultured in 5% CO_2_ at 37°C. Cells were confirmed to be mycoplasma-free by monthly testing using the MycoAlert Detection Kit (Lonza).

**Fluorescence-activated cell sorting (FACS).**

H295R and HEK293T cells were dissociated with trypsin-EDTA and resuspended in 20 ml of complete medium in T75 cell suspension flasks (Cellstar) overnight. The following day, cells were collected by centrifugation at 1000 × g for 5 minutes, resuspended in 5 ml medium, and passed through a 40 μm cell strainer before counting with a hemocytometer. Samples were divided into 1.5mL Eppendorf tubes (one unstained sample, one DAPI control, and one sorting sample). At least 50,000 cells were used for the unstained and DAPI controls, while the remaining cells were used for sorting. Tubes were spun at 1000 × g for 5 minutes. The supernatant was aspirated, and cells were resuspended in new tubes in 0.5 ml of sterile FACS buffer (50 mL PBS, 0.5 g bovine serum albumin (BSA), 2 mM EDTA). Tubes were centrifuged under the same conditions as above, and the supernatants were discarded. Cells were resuspended in 200 μL of FACS buffer, and the conjugated antibody (Human Pref-1/DLK1/FA1 Alexa Fluor® 488-conjugated Antibody, R&D Systems) was added to the sample for sorting at the recommended concentration of 5 μL/10^6^ cells. At the same time, 0.5 μL of the antibody was added to UltraComp eBeads™ Compensation Beads (Thermo Fisher) in 200 μL of FACS buffer. Samples were incubated on ice in the dark for 30 minutes, with vortexing every 10 minutes. All samples were washed 3 times with 1ml of FACS buffer and spun at 1000 × g for 5 minutes. DAPI was added to the single DAPI control and the sample for sorting at a final concentration of 0.1mg/mL solution. All samples were passed through a 40 μm cell strainer into polystyrene FACS tubes (Corning) and transported to the Flow Cytometry Facility in William Harvey Research Institute, QMUL. Staff in the facility optimized the settings and carried out the sorting as per departmental protocol using a BD FACSAria II. Gating was initially optimized using non-transfected and DLK1 transfected HEK293T cells. Sorted samples were collected in polystyrene FACS tubes (Corning) containing 0.5 ml of FACS buffer. DLK1^+^ and DLK1^-^ FACS-sorted H295R cells were immediately plated in 6 well-plates at a density of 3 x 10^3^ cells/well and cultured for 3 weeks, after which the number of colonies in each plate was manually counted. Sorted cells were also processed for RNA extraction.

**Spheroid generation**

Human H295R, CU-ACC1, MUC-1, TVBF7, and murine BCH-ACC3A cells were plated at 4-5 × 10^3^ cells per well in ultra-low attachment 6-well plates (Corning) in spheroid medium. The medium consisted of DMEM/Nutrient Mixture F-12 Ham (Sigma) supplemented with recombinant human basic fibroblast growth factor (20 ng/mL) (Sigma), recombinant human epidermal growth factor (20 ng/mL) (Sigma), B-27 (Thermo Fisher), and N-2 supplements (Thermo Fisher). Spheroids were allowed to form over 14 days (H295R, CU-ACC1, MUC-1, and TVBF7) or 7 days (BCH-ACC3A). Bright field images were taken with an AxiovertA1 microscope (Zeiss).

Medium was collected, centrifuged for steroid analysis, and total RNA was extracted from spheroids and 2D cultures for normalization. In parallel experiments, spheroids were allowed to settle by gravity in 15 ml tubes, washed with PBS, and lysed with RIPA buffer prior to western blotting.

**RNAScope**

Tissue sections were processed to detect mouse *Dlk1* (Red) and *Gli1* (Green) mRNAs using the RNAscope HD Duplex Reagent Kit, according to the manufacturer’s instructions (Supplementary Table S9).

***DLK1* isoforms PCR**

Primers were designed to simultaneously detect the full-length and shorter human *DLK1* isoforms: Forward (FW): 5’-AACAACAGGACCTGCGTGAG-3’; Reverse (REV): 5’-GCAGGTTCTTCTTCTTCCGCA-3’. Amplicon sizes were 754bp and 535bp, respectively. PCR was performed using New England Biolabs Hot Start *Taq* DNA Polymerase under the following cycling conditions: initial denaturation: 95°C for 30 seconds; 35 cycles of: 95°C for 20 seconds, 60°C for 30 seconds, 68°C for 30 seconds; final extension: 68°C for 5 minutes.

**Enzyme linked immunosorbence assay (ELISA) of patient serum/plasma**

In London, blood was taken from patients pre-operatively or at the start of chemotherapy treatment in the neo-adjuvant or non-operative management setting. Post-operative blood samples were taken at the first outpatient appointment. All blood draws included a yellow SST tube for serum collection. Samples were allowed to clot at RT for 10-15 minutes, then centrifuged at 1000 × g for 10 minutes at 4°C. Serum was aliquoted in 200-500 μL volumes and stored at -20°C. When possible, blood draws also included a purple EDTA tube for plasma collection, which was processed and stored following the same protocol.

In Germany, blood samples were processed and stored according to local protocols and guidelines. Samples were identified for analysis and aliquoted in 200 μL volumes for analysis. Serum and plasma samples were analyzed with the DLK1 Soluble (human) ELISA Kit (Adipogen) following the manufacturer’s instructions.

**Protein extraction**

Cells were washed in PBS and then lysed in cold RIPA lysis buffer (Thermo Fisher) supplemented with protease inhibitor cocktail (Roche). Lysates were kept on ice for 20 minutes and then cleared by centrifugation at 4°C for 10 minutes at 13,000 RPM. Protein concentration was determined using the BCA kit (Pierce).

**Western blotting**

Protein samples (20 μg) were size-separated on 4-12% NUPAGE gels (Thermo Fisher), and gels blotted onto nitrocellulose membranes (Protran). Membranes were stained with Ponceau to assess equal loading, de-stained in PBS containing 0.1% Tween-20 (PBS-T), blocked with 5% non-fat dry milk in PBS-T and incubated with primary antibody (Supplementary Table S10) overnight at 4°C. After washes in PBS-T, membranes were incubated with secondary antibodies (Supplementary Table S11). Immunoblots were scanned using the Odyssey FC Imaging System (LI-COR).

**RNA extraction and cDNA synthesis**

Total RNA was extracted using the RNeasy® Mini kit (Qiagen) according to the manufacturer’s instruction. Adrenals and ACC tissues were first dissociated with a blade and then minced using a Precellys 24 homogenizer (Bertin Instruments) with the Precellys Lysis kit in lysis buffer. During extraction, DNA was digested with DNaseI for 15 minutes at RT (Qiagen). RNA concentration and quality (A260/A280 ratio) was determined using a Nanodrop spectrophotometer (Thermo Fisher). A total of 500 ng of RNA in a 20 μl reaction was reverse transcribed into cDNA using the High-Capacity cDNA Reverse Transcription Kit (Applied Biosystems), according to the manufacturer’s instruction.

**Real Time qPCR**

Real-time quantitative PCR was performed using TaqMan® Universal Master Mix II and TaqMan® assays (Applied Biosystems, ABI). Pre-made primers and FAM-labeled probes were purchased from Thermo Fisher (Supplementary Table S12). The final reaction volume of 20 μL consisted of 10 μL TaqMan® Universal Master Mix II (2×), 1μL TaqMan® Gene Expression Assay (2×) and 9μL 2.5 ng/mL cDNA template. Amplification and detection were performed using the AriaMx Real-time PCR System under the following conditions: 95°C for 10 minutes, 40 cycles at 95°C for 10 seconds, and 60 °C for 1 minute. Each measurement was carried out in triplicate. Differences in gene expression, expressed as fold-change, were calculated using the 2^−ΔΔCt^ method, with *Gapdh* used as the internal control.

**GeoMx spatial transcriptomics**

Complete methods for GeoMx assays can be found in [6] and in the GeoMx manual. Four FFPE ACC samples were used for spatial transcriptomics. For each block, serial 5 μm sections were mounted onto Superfrost Plus Adhesion slides, and the best consecutive five were processed as follows: slide 1 for RNA quality control after scraping the sections off, slide 2 for H&E staining, slide 3 for DLK1 IHC, slide 4 as the experimental slide, and slide 5 for *DLK1* RNAScope. The experimental slides were backed in a 60°C drying oven for 1 hour, deparaffinized, and subjected to antigen unmasking in citrate buffer (pH 6.0) using a pressure cooker. Slides were then allowed to cool. A mix of Whole Transcriptome Atlas probes (WTA, Nanostring) was applied to each section and covered with HybriSlip Hybridization Covers. Slides were then incubated overnight for hybridization at 37°C in a Hyb EZ II hybridization oven (Advanced cell Diagnostics). The following day, HybriSlip covers were gently removed, and two 25-minutes stringent washes were performed in 50% formamide and 2X saline sodium citrate (SSC) at 37 °C. Slides were washed for 5 minutes in 2× SSC, then blocked in Buffer W (Nanostring) for 30 minutes at RT in a humidity chamber, washed in T-PBS, and blocked again in buffer W before overnight incubation in a humidity chamber at 4°C with anti DLK1 (B7, Santa Cruz Biotechnologies) diluted 1:100 in buffer W. After washes in 2X SSC, sections were incubated with secondary antibodies (Goat anti-mouse Alexa Fluor 488, Invitrogen, 1:500 dilution in buffer W) for 1 hour, and nuclei were stained with SYTO 13 (Nanostring). Sections were then loaded into a GeoMx DSP instrument, scanned, and 60 regions of interest (ROI) were selected based on DLK1 IF signal. The DLK1^+^ and DLK1^-^ ROI included only tumor cells, as determined by morphological and histological examination of the slide, along with adjacent H&E, RNAScope, and IHC slides.

ROI were then exposed to 385 nm UV light, allowing the release of indexing oligos, which were then collected in a 96-well plate. Oligos were dried and resuspended in 10 μL of DEPC-treated water. Sequencing libraries were generated by PCR from the photo-released indexing oligos, incorporating AOI-specific Illumina adapter sequences and unique i5 and i7 sample indices. Each PCR reaction contained 4 μL of indexing oligos, 1 μL of indexing PCR primers, 2 μL of Nanostring 5X PCR Master Mix, and 3 μL of PCR-grade water. Thermocycling conditions were: 37°C for 30 minutes, 50°C for 10 minutes, 95°C for 3 minutes; 18 cycles of 95°C for 15seconds, 65°C for 1minute, 68°C for 30 seconds; and 68°C 5 minutes. PCR reactions were pooled and purified twice using AMPure XP beads (Beckman Coulter, A63881) according to manufacturer’s protocol. Pooled libraries were sequenced at 2×75 base pairs and with the single-index workflow on an Illumina NextSeq to generate 458M raw reads. Data was analyzed using the Partek software (Illumina).

**Generation of H295R overexpressing DLK1 and with a knockdown of DLK1.**

H295R cells were transduced with lentiviral particles encoding human DLK1 (Precision LentiORF human DLK1 Tranduction starter kit, Horizon) or human DLK1 shRNA (SMARTvector Lentiviral shRNA, Horizon). Cells were selected with the appropriate antibiotic and tested for DLK1 protein expression using western blotting.

**RNA sequencing**

RNA was extracted from H295R grown at 70% confluency using the RNeasy® Mini kit (Qiagen) according to the manufacturer’s instructions. RNA quality and quantity were assessed using the Nanodrop 8000 and gel electrophoresis. Samples were sent to Eurofins Genomics (Germany) for library preparation and sequencing using strand-specific cDNA library and the Illumina HiSeq or NovaSeq 6000 S4 model (PE 150 XP).

Primary bioinformatic analysis was conducted on the DNA Nexus platform. Quality control of reads was performed using FastQC Reads Quality Control version 3.0.1. Read alignment was performed with HISAT2 version 1.03 [7], and differential expression analysis was performed using subread_featureCounts version 0.1.0 [8] and DESEQ2 (R.3.2-packages-quantification) [9]. Secondary analysis was performed using the WEB-based Gene Set Analysis Toolkit (WebGestalt) [10, 11] and the gene set enrichment analysis (GSEA) software [10] (https://www.gsea-msigdb.org/gsea/index.jsp). Differentially expressed gene (DEGs) were classified as those with an adjusted *P* < 0.05. Analysis of DEGs, and the generation of heatmaps and dot plots was performed using R Studio software (<https://posit.co/downloads/>). GSEA was performed using the R package fgsea, with gene rankings based on the Wald test statistic calculated by DESeq2. In addition to the MSigDB hallmark gene sets, a custom collection of adrenal-specific gene sets was constructed based on the adrenocortical differentiation score (ADS) [12], and the top differentially expressed genes in single-cell clusters from aged [13], cancerous [14] and fetal [15] adrenocortical tissue. DLK1 was excluded from these gene sets to prevent confounding results when comparing knockdown (KD) and overexpression (OE) cell lines. Comparisons of differential gene expression scores were visualized in heatmaps using the Wald test statistic. Pearson correlation coefficients for selected genes were calculated across TCGA ACC and normal adrenal gland GTEx samples using GEPIA [16].

**Steroid hormone quantification**

Quantification of steroids was performed by liquid chromatography-tandem mass spectrometry (LC-MS/MS).

*Calibrator, Internal Quality Control and Internal Standard solutions:* Certified reference material (Cerilliant, Merck) stock solutions were used for all analytes (each at 1000 mg/L, except pregnenolone, 11-deoxycorticosterone, cortisone and 21-deoxycortisol, which were at 100 mg/L). These stock solutions were used to prepare combined working solutions for calibration and internal quality control (IQC). To prepare these solutions, appropriate volumes of each stock solution were added to a glass tube and then dried down under nitrogen at 60°C. The steroids were then reconstituted in methanol to create calibrator and IQC working solutions, each containing: DHEAS (4000 μg/mL), cortisol (200 μg/mL), 17-hydroxypregnenolone (40 μg/mL) 17-hydroxyprogesterone (40 μg/mL), androstenedione (12 μg/mL), pregnenolone, corticosterone, 11-deoxycortisol, 21-deoxycortisol, cortisone (each at 40 μg/mL), testosterone (8 μg/mL) and 11-deoxycorticosterone (8 μg/mL). These working solutions were further diluted in methanol to create three additional working solutions: 3+20 (v/v), 1+39 (v/v) and 1:199 (v/v). All four working solutions were used to prepare calibration standards and IQC solutions by dilution in DMEM. After thorough mixing and equilibration (24 hours, 2–8°C), calibrators and IQC solutions were aliquoted into1.5 mL microcentrifuge tubes (Eppendorf, Stevenage, UK) and stored at -20 °C until use.

Internal standards (IS) stock solutions were prepared in methanol (each at 1000 mg/L). A combined IS sub-stock solution was also prepared in methanol, containing deuterated steroids at the following concentrations: DHEAS-D2 (75000 μg/mL), cortisol-D4 (15000 μg/mL), 17-hydroxypregnenolone-D3 (1500 μg/mL) 17-hydroxyprogesterone-D8 (500 μg/mL), androstenedione-D7 (150 μg/mL), pregenenolone-D4 (3000 μg/mL), cortisone-D2 and corticosterone-D8 (both at 1500 μg/mL), 11-deoxycortisol-D2 and 21-deoxycortisol-D8 (both at 200 μg/mL), testosterone-D3 (300 μg/mL) and 11-deoxycorticosterone-D8 (50 μg/mL). The IS working solution was freshly prepared before each batch by diluting 2.5 μL of IS stock per mL of methanol.

*Specimen processing:* Frozen calibrators, IQC solutions, and unknown media samples were thawed and mixed at room temperature (RT) by inversion before analysis. Aliquots (50 μL) of calibrator, IQC, and unknown media samples were transferred into 1.5 mL micro-centrifuge tubes, followed by the addition of 100 µL of IS working solution. Tubes were capped, vortex-mixed for 5 seconds, and then 200 μL of deionized water was added. After vortex-mixing for another 5 seconds, samples were centrifuged at 13,000 g for 5 minutes. A total of 300 μL of the supernatant was transferred into individual wells of an Oasis Max µElution solid phase extraction (SPE) plate (Waters Corp). Each SPE well had been preconditioned with 150 μL of methanol, followed by 150 μL of deionized water. Subsequently, each SPE well was washed with 100 µL of 1% (v/v) formic acid in 15% acetonitrile (aq), followed by 100 µL of 1% (v/v) ammonia in 15% acetonitrile (aq). Finally, captured steroids were eluted into a 96-well plate by adding 50 µL of 60% acetonitrile (aq) to each SPE well. 50 µL of deionized water was then added to each well of the collection plate.

*LC-MS/MS procedure:* LC-MS/MS was performed using a 1290 Infinity II LC System coupled with a 6495 triple quadrupole mass spectrometer (both Agilent Technologies). Extracts (5 μL) were injected onto an LC column (Zorbax Eclipse Plus C18 2.1x50mm, 1.8 µm) at a flow rate of 0.6 mL/min at 40°C. The mobile phases were: (A) 1 mmol/L ammonium fluoride in 60:40 (v/v) dH_2_O: methanol and (B) 1 mmol/L ammonium fluoride in methanol.
MS/MS was carried out in positive mode using electrospray ionization (ESI; Gas temp: 230°C, Gas Flow: 16 L/min, Nebulizer: 25 Psi, Sheath Gas Temp: 400°C, Sheath Gas Flow:12 L/min, Capillary: 4500 V, Nozzle voltage: 4500 V) operated in selected reaction monitoring (SRM) mode, with two m/z transitions per analyte and one m/z transition for each internal standard. LC-MS/MS instrument control, data acquisition, and post-analysis processing was performed using MassHunter (version B.09.00, Agilent Technologies). For assay calibration, peak area ratios (analyte quantifier to IS) were used to construct calibration graphs, with lines fitted by linear regression. The intercepts were not forced through zero, and line weighting (1/concentration) was applied. Deuterated ISs were used for all steroids in the developed method.

**Statistical analysis**

All data are presented as mean + standard deviation (SD) unless otherwise stated. Fisher’s exact test or the χ2 test was used to analyze dichotomic variables, while two-sided student’s t-test and Pearson correlation were used to test continuous variables. When multiple comparisons were made, a one-way ANOVA was performed, followed by post hoc Tukey’s multiple comparison tests to generate adjusted *p*-values. Correlations and 95% confidence intervals (Cis) between different parameters were evaluated by linear regression analysis. Overall survival (OS) was defined as the time from the date of primary surgery to disease-specific death or last follow-up, whereas recurrence-free survival (RFS) was defined as the time from the date of primary tumor resection, after complete resection (R0), to the first radiological evidence of any disease relapse or death. Progression-free survival (PFS) was defined as the time from the date of data capture to the first radiological evidence of disease progression, relapse, or death. All survival curves were generated using Kaplan–Meier estimates, and differences between survival curves were assessed with the log-rank (Mantel–Cox) test. Hazard ratios (HRs) were calculated by categorizing ACC cases into two groups based on DLK1 expression (higher or lower than median DLK1 expression). Additionally, four ACC groups were analyzed based on DLK1 expression quartiles (low, low-intermediate, high-intermediate, and high). A multivariate regression analysis was performed using the Cox proportional hazards regression model to identify factors that independently influenced survival. Statistical analyses were conducted using GraphPad Prism version 9.1 (La Jolla, CA, USA) and SPSS Software PASW Version 26.0 (SPSS, Inc., Chicago, IL, USA). *P*-values <0.05 were considered statistically significant.

**Supplementary Table S1.** **Descriptive characteristics of London patient cohort**

| **Clinicopathological feature** | | **Count** |
| --- | --- | --- |
| **Sex, *n* (%)** |  |  |
|  | Male | 26 (35.6) |
|  | Female | 47 (64.4) |
|  | Total | 73 (100) |
| **Age at operation (years), median (Range)** | | 52 (17-77) |
| **Diagnosis, *n* (%)** |  |  |
|  | ACC | 19 (26.0) |
|  | APA | 12 (16.4) |
|  | NAPACA | 28 (38.4) |
|  | Phaeochromocytoma | 1 (1.4) |
|  | Other benign | 9 (12.3) |
|  | Other malignant | 4 (5.5) |

Abbreviations: ACC, adrenocortical carcinoma; APA, aldosterone producing adenoma; NAPACA, non-aldosterone producing adrenocortical adenoma. The “other benign” category includes histological diagnoses of: micronodular hyperplasia, nodular and diffuse hyperplasia, ganglioneuroma, pseudocyst, focal nodular hyperplasia of zona glomerulosa, retroperitoneal schwannoma, perivascular epithelial cell tumor (PEComa), florid hyperplasia with myelolipomatous metaplasia, and a sample characterized by fibrotic material with calcification. The “other malignant” category includes liposarcoma, 2 metastases from adenocarcinoma of the lung, and a metastasis from ovarian carcinoma.

**Supplementary Table S2. Descriptive characteristics of tissue validation cohort (Würzburg)**

| **Clinicopathological feature** | | **Count** |
| --- | --- | --- |
| **Sex, *n* (%)** |  |  |
|  | Male | 53 (33.3) |
|  | Female | 105 (66) |
|  | Total | 159 (100) |
| **Age at operation (years), median (Range)** | | 47 (16-80) |
| **ENSAT tumor stage, *n* (%)** |  |  |
|  | I | 9 (5.1) |
|  | II | 79 (44.4) |
|  | III | 51 (28.7) |
|  | IV | 39 (21.9) |
| **Tumor entity, *n* (%)** |  |  |
|  | Primary | 131 (73.6) |
|  | Local recurrence | 27 (15.2) |
|  | Metastasis | 20 (11.2) |
|  | Total | 178 (100) |
| **Hormone secretion, *n* (%)** |  |  |
|  | Cortisol (alone or mixed) | 72 (40.4) |
|  | Other | 16 (9) |
|  | Inactive | 29 (16.3) |
| **Resection Status, *n* (%)** |  |  |
|  | 0 | 86 (48.3) |
|  | 1 | 14 (7.9) |
|  | 2 | 42 (23.6) |
|  | X | 36 (20.2) |
| **Weiss Score, median (Range)** | | 6 (2*-10) |
| **Ki-67%, median (Range)** | | 20 (1-90) |

* Weiss 2 ACC originally classified as ACA but then progressed

**Supplementary Table S3. Cox regression analyses of recurrence-free and progression-free survival in the validation cohort (Würzburg)**

| **Survival**  Variables | ***n*** | **Median survival (months)** | **Univariate** | | | **Multivariate** | | | |
| --- | --- | --- | --- | --- | --- | --- | --- | --- | --- |
|  |  |  | **HR** | **95% CI** | ***P*** | **HR** | **95% CI** | ***P*** |  |
| **RFS** | | | | | | | | |  |
| Age (years) |  |  |  |  |  |  |  |  |  |
| 0-49 | 51 | 18 |  |  |  |  |  |  |  |
| 50+ | 37 | 15 | 1.143 | 0.709 to 1.824 | 0.577 |  |  |  |  |
| ENSAT stage |  |  |  |  |  |  |  |  |  |
| I-II | 52 | 17 |  |  |  |  |  |  |  |
| III (+IV*) | 35 (36) | 14.3 | 1.174 | 0.729 to 1.874 | 0.577 |  |  |  |  |
| Ki-67% |  |  |  |  |  |  |  |  |  |
| 0-19 | 43 | 21 |  |  |  |  |  |  |  |
| 20+ | 42 | 10 | 1.832 | 1.130 to 2.991 | **0.014**** | 1.735 | 1.065 to 2.846 | **0.027*** |  |
| Glucocorticoid excess |  |  |  |  |  |  |  |  |  |
| Absent | 29 | 18 |  |  |  |  |  |  |  |
| Present | 34 | 13 | 1.334 | 0.768 to 2.348 | 0.309 |  |  |  |  |
| DLK1 expression |  |  |  |  |  |  |  |  |  |
| Low | 40 | 22.5 |  |  |  |  |  |  |  |
| High | 48 | 10.5 | 1.952 | 1.210 to 3.200 | **0.007**** | 1.791 | 1.097 to 2.966 | **0.021*** |  |
| **PFS** | | | | | | | | |  |
| Age (years) |  |  |  |  |  |  |  |  |  |
| 0-49 | 97 | 9 |  |  |  |  |  |  |  |
| 50+ | 79 | 7 | 1.094 | 0.794 to 1.504 | 0.579 |  |  |  |  |
| ENSAT stage |  |  |  |  |  |  |  |  |  |
| I-II | 57 | 17 |  |  |  |  |  |  |  |
| III | 44 | 11 | 1.236 | 0.801 to 1.897 | 0.334 | 2.134 | 1.231 to 3.711 | **0.007**** |  |
| IV | 75 | 5 | 2.245 | 1.539 to 3.307 | **< 0.001****** | 1.783 | 0.968 to 3.284 | 0.063 |  |
| Ki-67% |  |  |  |  |  |  |  |  |  |
| 0-19 | 68 | 15 |  |  |  |  |  |  |  |
| 20+ | 75 | 7 | 1.635 | 1.142 to 2.350 | **0.007**** | 1.786 | 1.133 to 2.850 | **0.013*** |  |
| Glucocorticoid excess |  |  |  |  |  |  |  |  |  |
| Absent | 44 | 14.5 |  |  |  |  |  |  |  |
| Present | 73 | 6 | 1.494 | 0.999 to 2.269 | 0.054 | 1.280 | 0.798 to 2.070 | 0.309 |  |
| Resection status |  |  |  |  |  |  |  |  |  |
| 0 & X | 123 | 10 |  |  |  |  |  |  |  |
| 1 & 2 | 53 | 5 | 2.36 | 1.656 to 3.325 | **< 0.001****** | 2.260 | 1.298 to 3.886 | **0.004**** |  |
| DLK1 expression |  |  |  |  |  |  |  |  |  |
| Low | 88 | 8 |  |  |  |  |  |  |  |
| High | 88 | 7 | 1.33 | 0.966 to 1.834 | 0.081 | 1.489 | 0.956 to 2.331 | 0.079 |  |

**Supplementary Table S4. Descriptive characteristics of validation cohort for serum analysis (Würzburg)**

| **Clinicopathological feature** | | **Count** |
| --- | --- | --- |
| **Sex, *n* (%)** |  |  |
|  | Male | 8 (32) |
|  | Female | 17 (68) |
|  | Total | 25 (100) |
| **Age at operation sample (years), median (Range)** | | 51 (26-80) |
| **Disease status at time of sample, *n* (%)** |  |  |
|  | Primary tumor | 9 (23.1) |
|  | Primary tumor and metastases | 6 (15.4) |
|  | Recurrence after primary surgery | 17 (43.6) |
|  | Disease free | 7 (17.9) |
|  | Total | 39 (100) |
| **Hormone secretion, *n* (%)** |  |  |
|  | Cortisol (alone or mixed) | 12 (50) |
|  | Androgens | 2 (8.3) |
|  | Inactive | 8 (33.3) |
|  | Unknown | 2 (8.3) |
| **ENSAT tumor stage at time of sample, *n* (%)** |  |  |
|  | I | 0 (0) |
|  | II | 3 (10.7) |
|  | III | 3 (10.7) |
|  | IV | 22 (78.6) |
| **Tumor size (cm) , *n* (%), median (Range)** |  |  |
|  | Primary tumor | 15 (83.3), 13 (6.5-19) |
|  | Local recurrence | 3 (16.7), 4 (3.5-7) |
| **Resection status, *n* (%)** |  |  |
|  | 0 | 7 (70) |
|  | 1 | 2 (20) |
|  | 2 | 5 (50) |
|  | X | 2 (20) |
|  | N/A | 3 (30) |
| **Weiss score, median (Range)** | | 8 (5-10) |
| **Ki-67 %, median (Range)** | | 20 (10-70) |

**Supplementary Table S5. LC-MS/MS hormonal output of different ACC cell lines grown in adherent and spheroid culture**

| **Cell line** | **Steroid** | **2D** | | | | **3D** | | | | **Significance** | **P** |
| --- | --- | --- | --- | --- | --- | --- | --- | --- | --- | --- | --- |
|  |  | **Mean normalized concentration (nmol/L/RNA mass) ± SD** | | | ***n*** | **Mean normalized concentration (nmol/L/RNA mass) ± SD** | | | ***n*** |  |  |
| **H295R** | Corticosterone | 0.000 | ± | 0.000 | 3 | 1.485 | ± | 0.404 | 3 | ** | 0.003 |
|  | 11-deoxycortisol | 6.170 | ± | 1.609 | 3 | 14.960 | ± | 3.890 | 3 | * | 0.022 |
|  | Androstenedione | 4.800 | ± | 1.349 | 3 | 1.934 | ± | 0.589 | 3 | * | 0.028 |
|  | 11-deoxycorticosterone | 1.404 | ± | 0.329 | 3 | 29.43 | ± | 5.611 | 3 | *** | 0.001 |
|  | 17-hydroxyprogesterone | 11.68 | ± | 3.650 | 3 | 3.510 | ± | 0.137 | 3 | * | 0.018 |
|  | Progesterone | 0.827 | ± | 0.193 | 3 | 3.474 | ± | 0.289 | 3 | *** | < 0.001 |
| **CU-ACC1** | Cortisone | 51.69 | ± | 13.05 | 3 | 26.24 | ± | 1.050 | 3 | * | 0.028 |
|  | Cortisol | 18.83 | ± | 5.901 | 3 | 311.4 | ± | 84.42 | 3 | ** | 0.004 |
|  | Corticosterone | 1.052 | ± | 0.279 | 3 | 61.10 | ± | 32.75 | 3 | * | 0.034 |
|  | 11-deoxycortisol | 13.67 | ± | 3.38 | 3 | 3.31 | ± | 1.15 | 3 | ** | 0.007 |
|  | Androstenedione | 5.57 | ± | 1.37 | 3 | 1.52 | ± | 0.5 | 3 | ** | 0.009 |
|  | 11-deoxycorticosterone | 0.53 | ± | 0.11 | 3 | 0.36 | ± | 0.18 | 3 | ns | 0.235 |
|  | Testosterone | 1.22 | ± | 0.28 | 3 | 0.18 | ± | 0.06 | 3 | ns | 0.281 |
|  | 17-hydroxyprogesterone | 1.67 | ± | 0.4 | 3 | 1.06 | ± | 0.42 | 3 | ns | 0.141 |
|  | Progesterone | 0.000 | ± | 0.000 | 3 | 0.196 | ± | 0.095 | 3 | * | 0.024 |
| **MUC1** | 11-deoxycortisol | 0.00 | ± | 0.00 | 3 | 0.81 | ± | 0.69 | 3 | ns | 0.111 |
|  | Androstenedione | 0.20 | ± | 0.03 | 3 | 0.64 | ± | 0.63 | 3 | ns | 0.295 |
|  | 17-hydroxyprogesterone | 0.43 | ± | 0.11 | 3 | 1.30 | ± | 1.39 | 3 | ns | 0.340 |
|  | Cortisone | 0.35 | ± | 0.20 | 3 | 2.96 | ± | 4.09 | 3 | ns | 0.332 |
| **TVBF7** | 11-deoxycortisol | 0.00 | ± | 0.00 | 3 | 0.29 | ± | 0.51 | 3 | ns | 0.374 |
|  | Androstenedione | 0.04 | ± | 0.02 | 3 | 0.34 | ± | 0.39 | 3 | ns | 0.258 |
|  | 17-hydroxyprogesterone | 0.20 | ± | 0.08 | 3 | 1.36 | ± | 0.77 | 3 | ns | 0.060 |
| **BCH-ACC3A** | Corticosterone | 111.19 | ± | 35.93 | 4 | 759.57 | ± | 80.84 | 3 | **** | < 0.001 |
|  | 11-deoxycortisol | 9.06 | ± | 3.11 | 4 | 57.02 | ± | 5.32 | 3 | **** | < 0.001 |
|  | 11-deoxycorticosterone | 7.23 | ± | 2.38 | 4 | 11.39 | ± | 1.87 | 3 | ns | 0.056 |

**Supplementary Table S6. List of primary antibodies used for IHC and IF**

| **Primary antibodies (IHC and IF)** | **Host** | **Source** | **Identifier** | **Dilution** | **AUM** |
| --- | --- | --- | --- | --- | --- |
| CYP11B2 (AS 2084) | Rabbit | Celso Gomez-Sanchez, University of Mississippi, USA | N/A | 1:100 | Yes |
| DLK1 (B7) | Mouse | Santa Cruz | Sc-376755 | 1:100 | Yes |
| RFP | Rabbit | Antibodies-online | ABIN129578 | 1:100 | Yes |
| RFP | Goat | Sicgen | AB8181-200 | 1:200 | Yes |
| SF1/NR5A1 (N1665) | Mouse | Invitrogen | 434200 | 1:100 | Yes |
| GATA4 (C-20) | Rabbit | Santa Cruz | sc-1237 | 1:150 | No |
| Tyrosine Hydroxylase | Rabbit | Millipore | AB152 | 1:100 | Yes |
| Ki67 | Rabbit | Abcam | Ab15580 | 1:200 | Yes |
| GFP | Chicken | Abcam | Ab13970 | 1:400 | Yes |
| Active β-catenin | Rabbit | Cell Signaling | 4270S | 1:100 | Yes |
| Cleaved caspase 3 | Rabbit | Abcam | Ab2302 | 1:100 | Yes |

**Supplementary Table S7. List of secondary antibodies used for IHC**

| **Secondary Antibodies (IHC)** | **Host** | **Source** | **Identifier** | **Dilution** |
| --- | --- | --- | --- | --- |
| Biotinylated anti Mouse IgG | Goat | Vector | BA-9200 | 1:500 |
| Biotinylated anti Goat IgG | Horse | Vector | BA-9500 | 1:500 |
| Biotinylated anti Rabbit IgG | Goat | Vector | BA-1000 | 1:500 |

**Supplementary Table S8. List of secondary antibodies used for IF**

| **Secondary Antibodies (IF)** | **Host** | **Source** | **Identifier** | **Dilution** |
| --- | --- | --- | --- | --- |
| Alexa Fluor 488 anti-Chicken IgG | Goat | Invitrogen | A11039 | 1:1000 |
| Alexa Fluor 568 anti-Mouse IgG | Goat | Invitrogen | A11004 | 1:1000 |
| Alexa Fluor 488 anti-Rabbit IgG | Goat | Invitrogen | A11008 | 1:1000 |
| Alexa Fluor 568 anti-Rabbit IgG | Goat | Invitrogen | A11036 | 1:1000 |
| Alexa Fluor 488 anti-Mouse IgG | Goat | Invitrogen | A11029 | 1:1000 |

**Supplementary Table S9. List of RNAScope reagents and probes**

| **RNAScope reagents and probes** | Source | Identifier |
| --- | --- | --- |
| RNAscope® 2.5 High Definition (HD)- RED Assay | ACD | 322350 |
| RNAscope® HD Duplex Reagent Kit | ACD | 322430 |
| RNAscope® Probe - Mm-Gli1 - Mus musculus GLI-Kruppel family member GLI1 (Gli1), mRNA | ACD | 31 1001 |
| RNAscope® Probe - Mm-Dlk1-C2 - Mus musculus delta-like 1 homolog (Drosophila) (Dlk1) transcript 1 variant 2 mRNA | ACD | 405971-C2 |

**Supplementary Table S10. List of primary antibodies used in Western Blotting**

| **Primary antibodies (Western Blotting)** | **Host** | **Source** | **Identifier** | **Dilution** |
| --- | --- | --- | --- | --- |
| DLK1 (B7) | Mouse | Santa Cruz | sc-376755 | 1:100 |
| GAPDH | Mouse | Santa Cruz | sc-47724 | 1:2000 |

**Supplementary Table S11. List of secondary antibodies used in Western Blotting**

| **Secondary antibodies (Western Blotting)** | **Host** | **Source** | **Identifier** | **Dilution** |
| --- | --- | --- | --- | --- |
| IRDye 680 RD anti mouse IgG | Goat | Li-cor | 926-68070 | 1:10000 |

**Supplementary Table S12. List of TaqMan Probes used for Real Time qPCR**

| **TaqMan Probes (FAM)** | Source | Identifier |
| --- | --- | --- |
| *GAPDH* human | Thermo Fisher | Hs99999905 |
| *DLK1* human | Thermo Fisher | Hs00171584 |


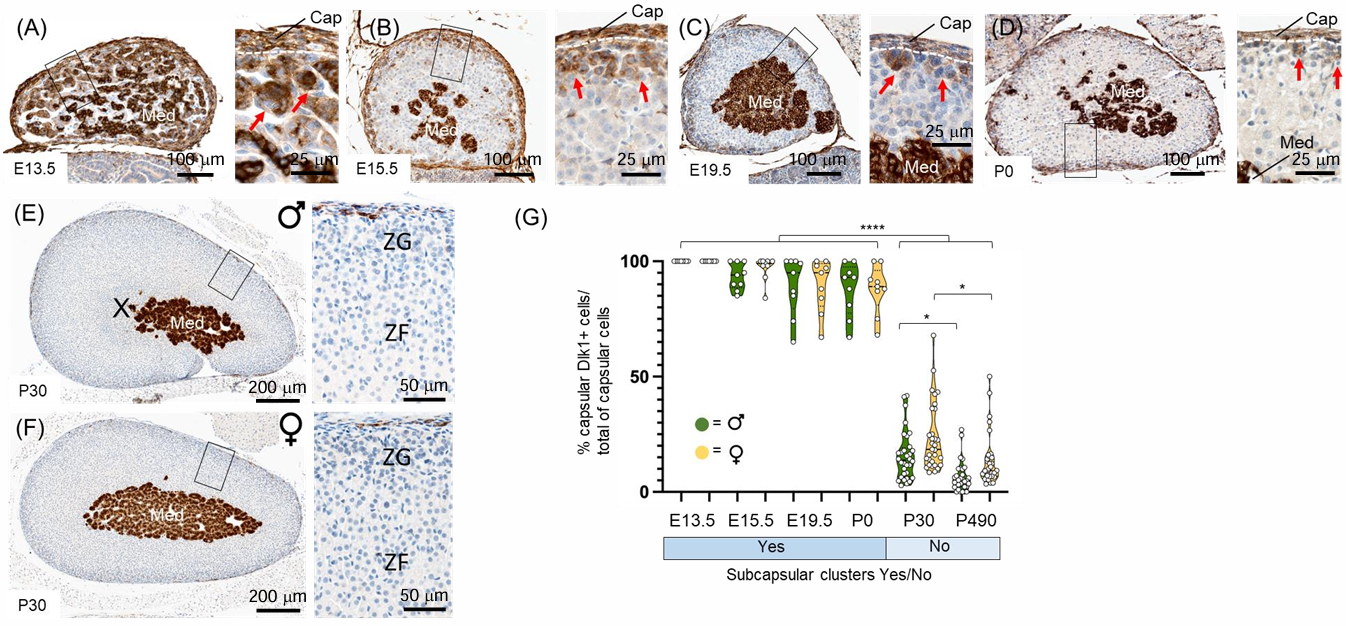


**Supplementary Figure S1. Embryonic and postnatal expression of Dlk1 in the mouse adrenal.**

**(A-D)** Immunohistochemical detection of Dlk1 in E13.5 (A), E15.5 (B), and E19.5 (C) adrenals, showing expression in the capsule, cortex, and medulla. Virtually all capsular cells displayed Dlk1 immunoreactivity up to E15.5 and maintained high expression up to P0. Subcapsular clusters of Dlk1^+^ cells (red arrows) decreased during development and were sparse at P0 (D).

(E-F) Immunohistochemical detection of Dlk1 in 4-weeks old female (E) and male (F) adrenals. High expression of Dlk1 in the medulla was detected at all stages analyzed.

(G) The percentage of Dlk1^+^ cells in the capsule dramatically decreased after birth, with a small, non-significant trend of a higher number of Dlk1^+^ cells in female mice.

Abbreviations: Cap, capsule; E, embryonic day; P, postnatal day; ZG, Zona Glomerulosa; ZF, Zona Fasciculata; Med, medulla; X, X-zone. * *P* < 0.05, **** *P* < 0.0001.

**
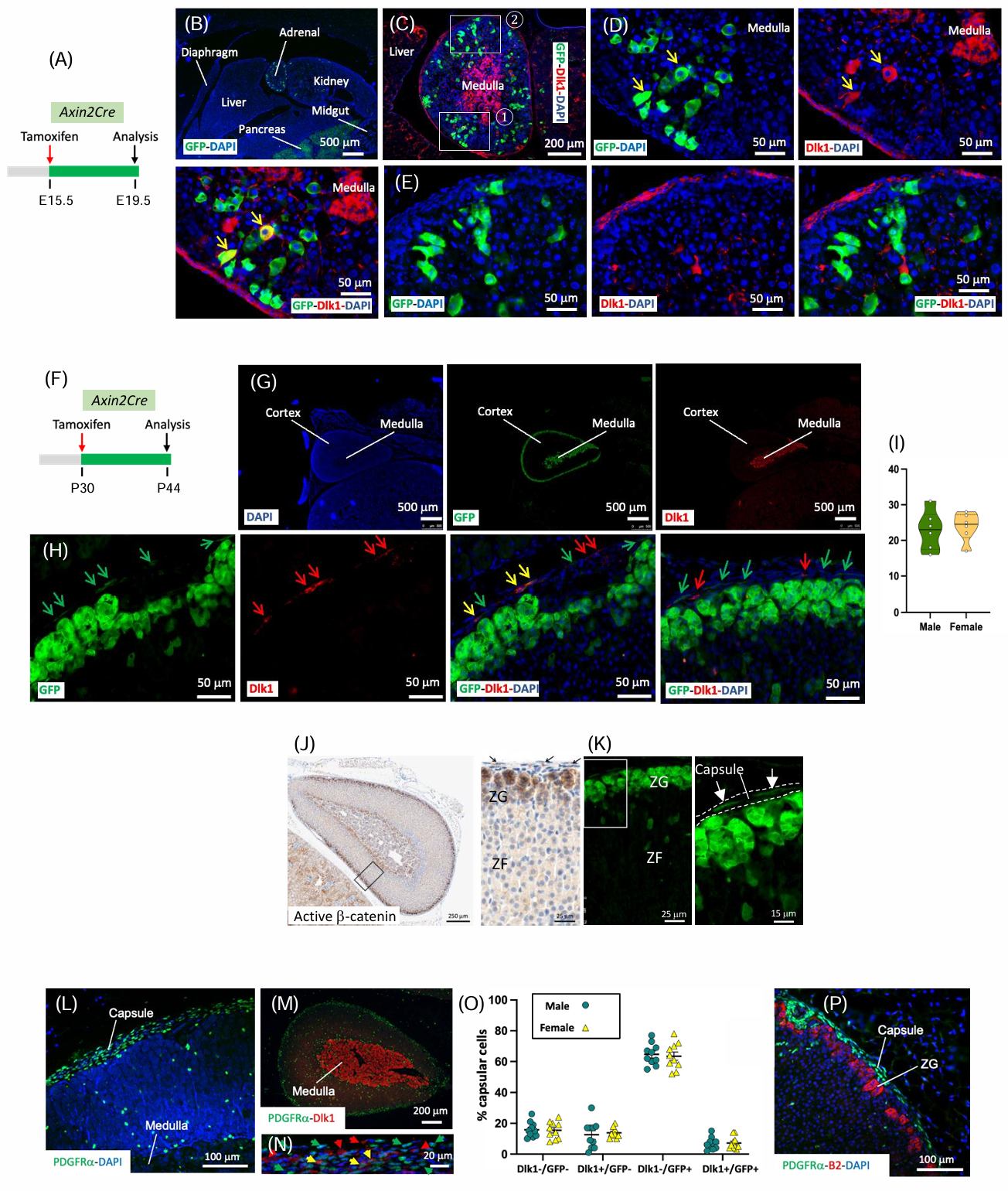
**

**Supplementary Figure S2. Spatial relationship between Dlk1-expressing cells, subcapsular Axin2^+^ early progenitors, and PDGFRα^+^ capsular cells in postnatal mice.**

(A) *Axin2^CreERT2/+^;Rosa^YFP/YFP^* mice (*Axin2Cre*) were employed to determine the spatial relationship between Dlk1-expressing cells and subcapsular Axin2^+^ early adrenal progenitor cells [17]. *Axin2Cre* mice express inducible Cre recombinase in Axin2^+^ cells, and tamoxifen-induced recombination at the Rosa26 locus results in the permanent labelling of Axin2-expressing cells and their progeny with yellow fluorescence protein (YFP). YFP^+^ cells were detected with an anti-GFP antibody. The schematic illustrates the time of fate mapping in tamoxifen-treated dams.

(B-E) Localization of Axin2^+^ cells and their early progeny (4-day chase, green) relative to Dlk1 expression (red). Nuclei are stained with DAPI (blue). (D) corresponds to ① in (C) and (E) corresponds to ② in (C). Occasional cortical GFP^+^/Dlk1^+^ cells (yellow arrows in D) were observed, while the capsule, which strongly expressed Dlk1, lacked GFP staining. GFP^+^/Dlk1^+^ cells were negative for tyrosine hydroxylase (TH) and steroidogenic factor 1 (Sf1) (data not shown).

(F) Schematic of tamoxifen treatment in postnatal *Axin2Cre*.

(G-H) Localization of Axin2^+^ cells and their early progeny (14-day chase, green) relative to Dlk1 expression (red). Capsular GFP^+^/Dlk1^-^ cells are indicated with green arrows, GFP^+^/Dlk1^+^ cells with yellow arrows, and Dlk1^+^/GFP^-^ cells with red arrows.

(I) Percentage of GFP^+^ cells within the capsular Dlk1^+^ population in male and female adrenals.

(J-K) The potential presence of capsular Wnt activity was further investigated through immunohistochemical staining for active β-catenin (J), which showed classical subscapular staining with occasional spindle-like capsular cells. This pattern was comparable to the GFP signal observed in aged-matched *Axin2Cre* mice (K, 14 days chase).

(L-N) Immunofluorescence staining of adrenals from PDGFRα^EGFP^ mice, which express a histone H2B- eGFP fusion protein from the endogenous *PDGFRα*locus and the GFP signal is therefore nuclear; PDGFRα (CD140b) marks mesenchymal stem cells/fibroblastic cells [18]. Sections stained for GFP (indicating PDGFRα^EGFP^ cells) showed strong capsular staining (L). Staining for GFP and Dlk1 (M) revealed colocalization patterns, with panel (N) providing a high-magnification view of the capsule: green arrows indicate PDGFRAα^+^/Dlk1^-^ cells, red arrows indicate Pdgfrα^−^/Dlk1^+^ cells, and yellow arrows indicate PDGFRα^+^/Dlk1^+^ cells.

(O) While most PDGFRα^+^cells were negative for Dlk1 expression, approximately 5% were double positive in both males and females.

(P) As expected, PDGFRα^+^ cells were adjacent to, but distinct from, Cyp11b2-expressing ZG cells (B2, red).

Abbreviations: E, embryonic day; P, postnatal day; YFP, yellow fluorescent protein.


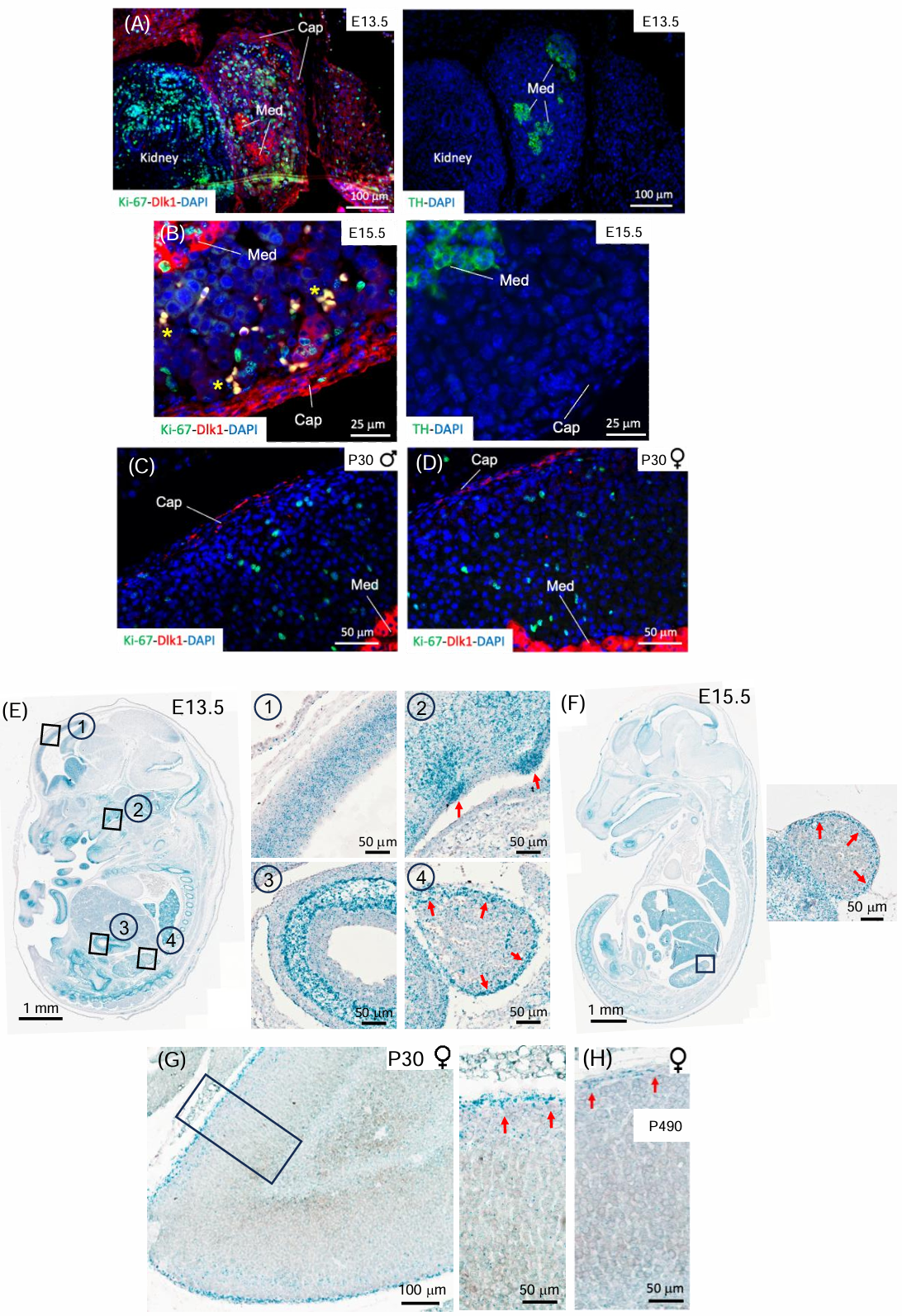


**Supplementary Figure S3. Dlk1^+^ cells were rarely proliferative, and *Gli1* capsular expression remained high throughout postnatal life.**

(A-D) Dlk1^+^ cells rarely proliferated. Representative immunofluorescence staining for Ki-67, Dlk1, and DAPI in E13.5 (A), E15.5 (B) and P30 (C and D) adrenals, showing extremely rare colocalization of Ki-67 with Dlk1 in the capsule (<1%) and in subcapsular clusters (<5%) during development, with no colocalization postnatally. The developing medulla was identified usingTyrosine Hydroxylase (TH) antibodies (A and B) in adjacent sections. Yellow asterisks (B) indicate autofluorescent erythrocytes.

(E-H) Strong capsular expression of *Gli1* was maintained throughout the lifespan of mice. RNAScope detection of *Gli1* mRNA at E13.5 (E), E15.5 (F), P30 (G), and P490 (H), showing strong staining in the capsule during development (E, F) and persisting into old age (G-H). Expression of *Gli1* in male adrenals was similar (not shown). Known areas of *Gli1* expression are shown in panels (E) ① (neocortex), (E) ② (mesenchyme by the papillae, arrows), and (E) ③ (mesenchyme by the stomach).

Abbreviations: Cap, capsule; E, embryonic day; Med, medulla; P, postnatal day; TH, Tyrosine Hydroxylase.

**
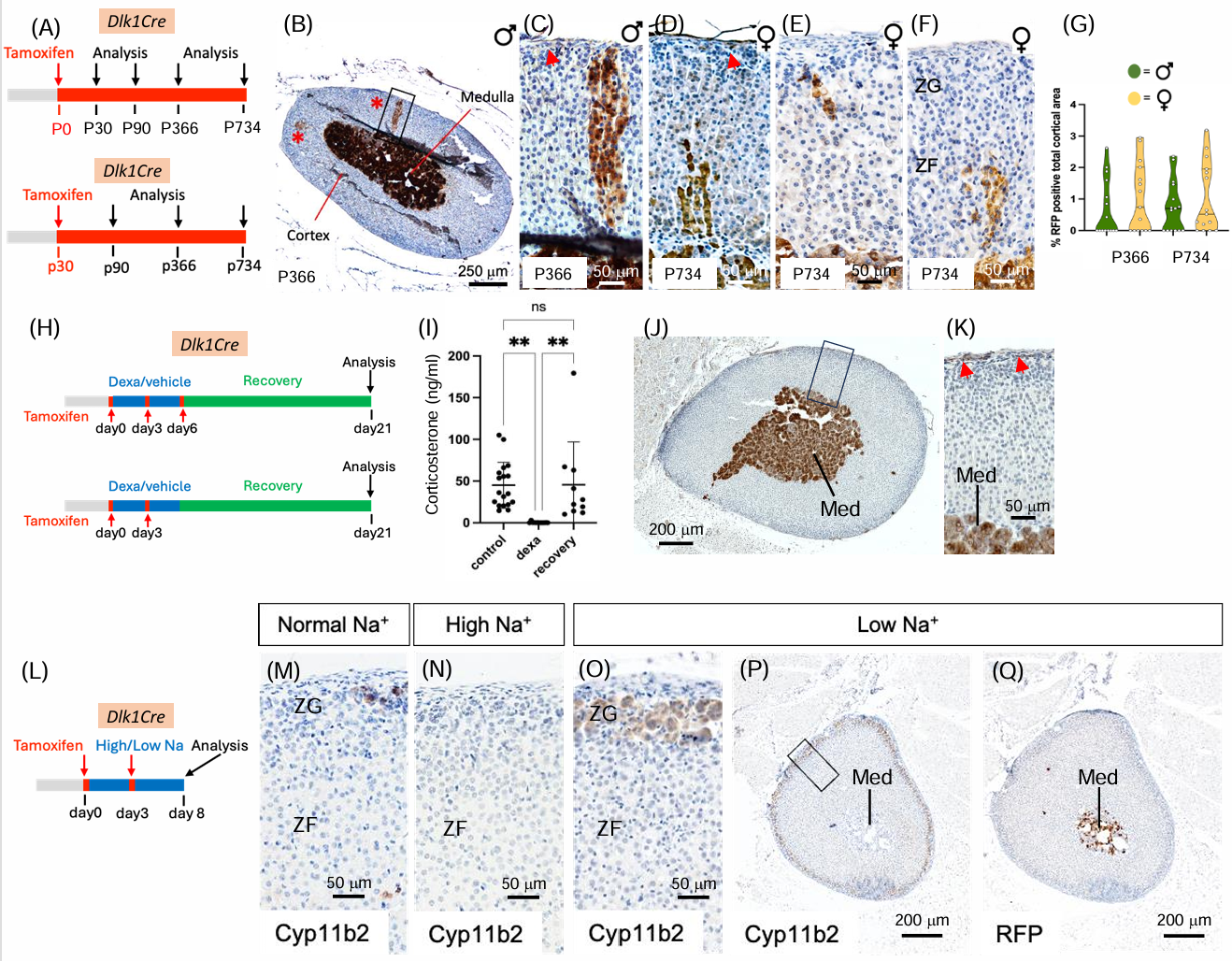
**

**Supplementary Figure S4. Capsular Dlk1^+^ cells were near-dormant postnatally and remained inactive upon adrenocortical remodeling.**

(A) Schematics of tamoxifen induction in P0 and P30 *Dlk1Cre* mice. Injection of tamoxifen in males and females at P0 and P30, followed by chase periods of 2 weeks, 1 months, 2 months, and 3 months, did not result in detectable cortical RFP^+^ cells (data not shown) in either sex.

(B-F) Representative immunohistochemical detection of RFP^+^ cells after a one-year chase in male (B-C) and a two-years chase in female (D-F) mice. Red asterisks in the panoramic panel (B) indicate occasional clusters and columns of RFP^+^ cells. These cortical RFP^+^ cells were TH^-^ (data not shown). Red arrows indicate capsular RFP^+^ cells.

(G) Time-course quantification of RFP^+^ cells in the cortex.

(H) Schematics of tamoxifen induction and dexamethasone treatment in P30 and P460 *Dlk1Cre* mice.

(I) Serum corticosterone levels measured before dexamethasone treatment (control, at P30), after the dexamethasone regimen (dexa), and after zona fasciculata (ZF) regeneration (recovery). Vehicle-treated mice did not show changes in corticosterone levels (data not shown).

(J-K) Immunohistochemical detection of RFP^+^ cells after ZF regeneration in *Dlk1Cre* mice showed capsular and medullary RFP^+^ cells, but not cortical RFP^+^ cells (indicating a static Dlk1 population, contrasts with the reported reactivation of capsular Gli1^+^ cells during ZF regeneration [19]). Similar results were obtained in P460 *Dlk1Cre* mice (data not shown).

(L) Schematic of tamoxifen induction of P50 and P70 *Dlk1Cre* mice. ZG remodeling was induced by dietary sodium manipulation: a low-sodium (Na^+^) diet to expand the zona glomerulosa (ZG) and a high-Na^+^ diet to induce ZG regression.

(M-P) Representative immunohistochemical detection of Cyp11B2 expression in P50 female mice fed with normal (M), high (N), and low Na^+^ (O, P) diets, showed expected changes in Cyp11B2 expression, consistent with differential aldosterone requirements.

(Q) Immunohistochemical detection of RFP^+^ cells in P50 female mice after a low Na^+^ diet, showing no RFP immunoreactivity in the ZG or elsewhere in the cortex. Similar results were observed in males, and in P70 females and males (data not shown). No RFP expression was detected in the ZG or cortex of tamoxifen-injected P50/P70 *Dlk1Cre* mice in the high Na^+^ groups (data not shown). The strong RFP signal in the medulla in (B-F, J, K, Q) confirmed efficient recombination.

Abbreviations: E, embryonic day; P, postnatal day; Dexa, dexamethasone treated; RFP, red fluorescence protein; Med, medulla; ZG, zona glomerulosa; ZF, zona fasciculata; TH, tyrosine hydroxylase. ***P* < 0.01; ns, not significant.

**
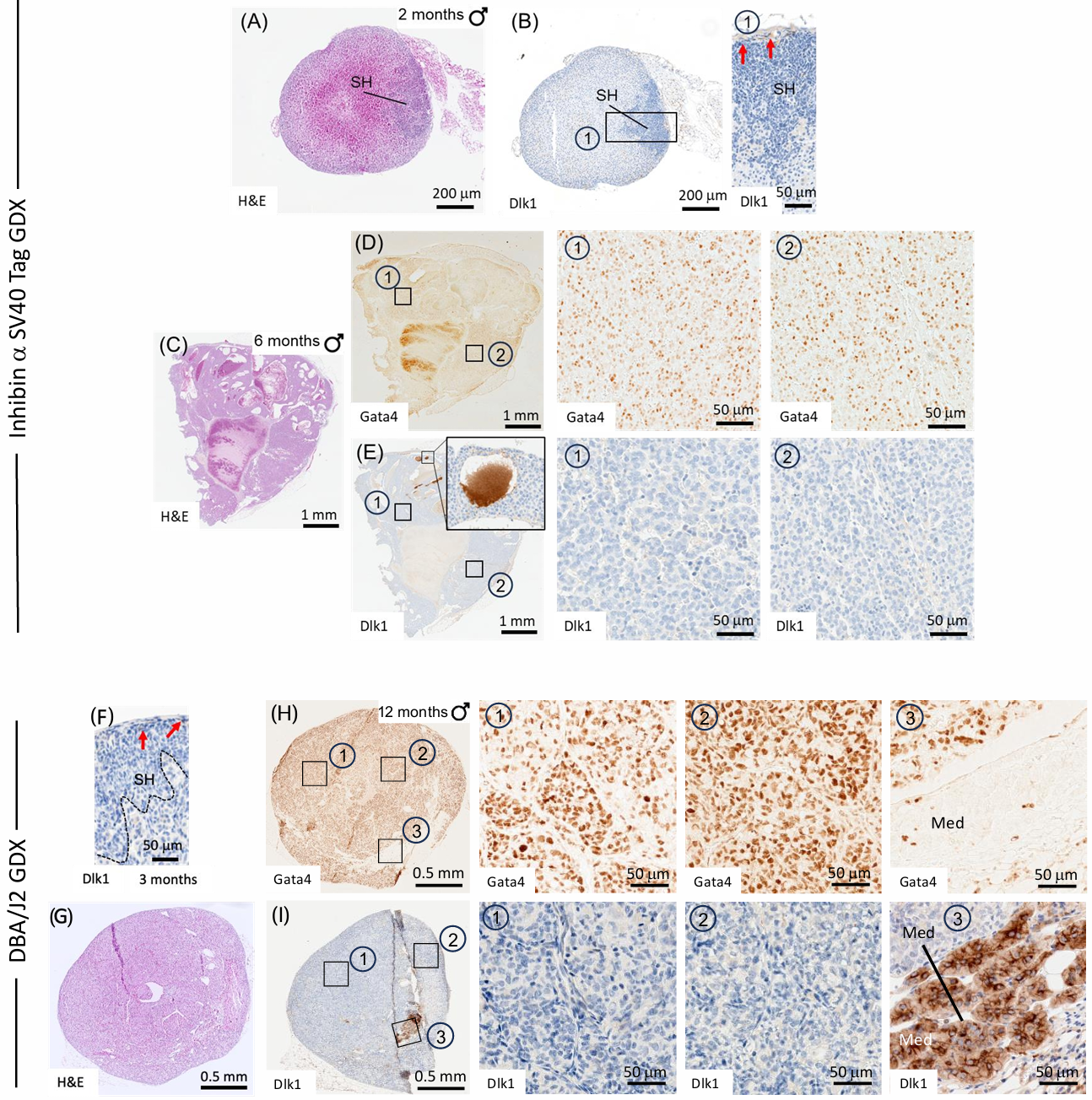
**

**Supplementary Figure S5.** **Dlk1 was not expressed in adrenal subcapsular hyperplasia (SH) or subsequent adrenocortical tumors in mice.**

(A-E) Inhibin α-SV40 Tag mice underwent prepubertal gonadectomy (GDX), resulting in the development of SH at 2 months (A, B), followed by adrenocortical tumors at 6 months (C-E). Expression of Dlk1 was restricted to the external capsule in SH (red arrows in B) and was absent in tumors (E). Tumors strongly expressed the Gata4 transcription factor (D). The magnified panel in (E) shows artefactual staining in luminal secretions.

(F-I) DBA/2Jmice underwent prepubertal gonadectomy (GDX), resulting in the development of SH at 3 months (F), followed by adrenocortical tumors at 12 months (G-I). Expression of Dlk1 was restricted to the external capsule in SH (red arrows in F) and was absent in tumors (I), except in remnants of the medulla (Med, panel ③). Tumors strongly expressed the Gata4 transcription factor (H).

Abbreviations: GDX, gonadectomy; Med, medulla; SH, subcapsular hyperplasia.


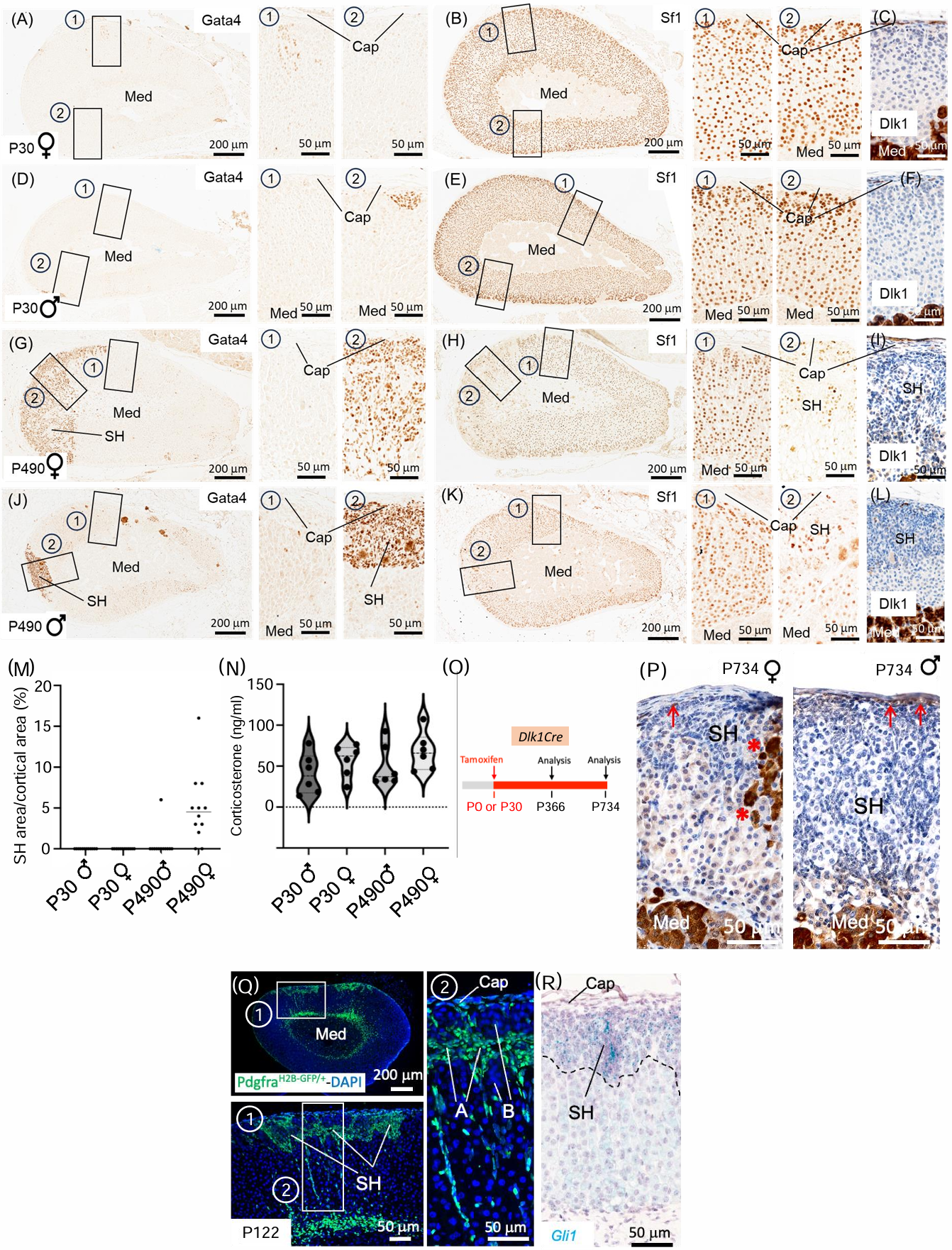


**Supplementary Figure S6.** **SH foci in aged mice were not enriched in, nor derived from, Dlk1-expressing cells.**

(A-L) Expression of Gata4, Sf1, and Dlk1 in young (P30) and aged (P490) adrenals. Large SH foci were observed in 11/12 females and in 1/12 males. Panels (J-K) are not representative of male adrenals but show the only male that developed SH. Dlk1 expression (panels C, F, I, and L) was restricted to the outermost part of each SH, while both SH A and SH B cells were negative for Dlk1 expression.

(M) Percentage area of SH, expressed as the ratio of Gata4^+^ cells to the total cortical area.

(N) LC-MS/MS quantification of corticosterone levels in the sera of P30 and P490 mice.

(O) Schematic of tamoxifen induction in *Dlk1Cre* mice.

(P) A total of 57 SH foci from 24 mice (3 males and 3 females per condition; injection at P0 and P30, with chase periods up to P366 and P734) were analyzed for RFP expression. None of the SH foci were RFP-positive, with RFP^+^ cells confined to the outermost part of the SH (red arrows), the medulla, and occasional non-SH cluster/columns representing Dlk1 cortical steroidogenic progeny (red asterisks in the left panel).

(Q-R) SH A cells (spindle-shaped and capsular-like cells within SH) were PDGFRα^+^ and *Gli1^+^*, suggesting the contribution of distinct capsular fibroblast-like populations to SH formation. SH B cells (nests of larger, lipid-laden polygonal cells within SH) were negative for these markers.

Abbreviations: Cap, capsule; Med, medulla; P, postnatal day; SH, subcapsular hyperplasia; RFP, red fluorescence protein.


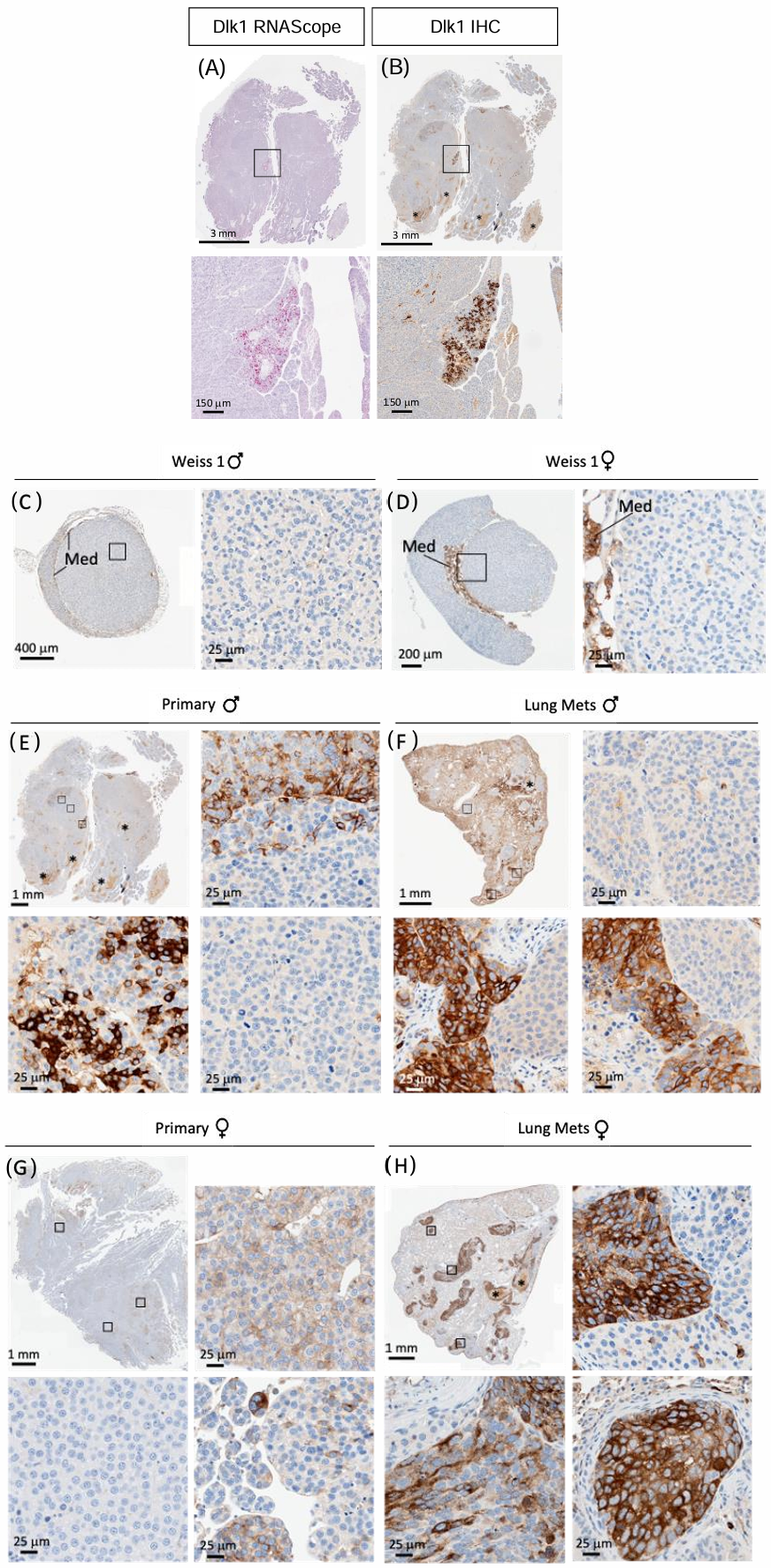


**Supplementary Figure S7. Dlk1 was re-expressed in a murine model of ACC and exhibited intratumoral heterogeneity.**

(A-B) Examples of *Dlk1* RNA (A) and Dlk1 protein (B) expression in a low H-score ACC from *BPCre* mice (consecutive sections), showing both negative and positive areas within the tumor parenchyma. The expression patterns of *Dlk1* mRNA and protein were complementary.

(C-H) Immunohistochemical detection of Dlk1 expression in ACC from *BPCre* male (C) and female (D) mice with a low Weiss Score, in metastatic ACC (E, primary tumor in males; G, primary tumor in females), and in lungs metastasis (Mets: F, males; H, females). Dlk1 expression was higher in metastatic ACC and lungs metastasis, displaying both a diffuse and a clustered (or even clonal) pattern. Note that top left panel in (H) is also shown in Figure 1H.

Abbreviations: ACC, adrenocortical carcinoma; IHC, immunohistochemistry; Med, medulla; Mets, metastasis.


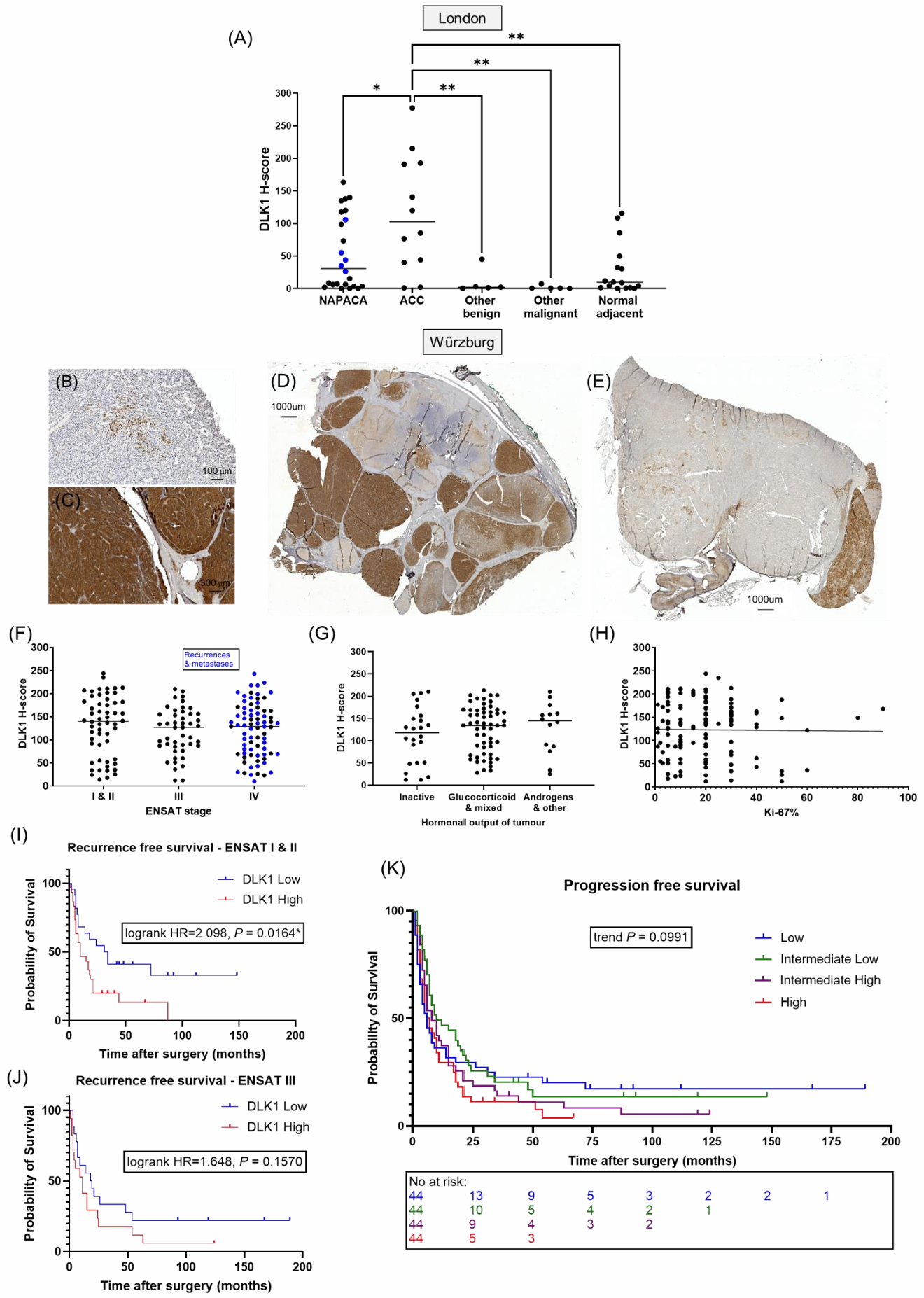


**Supplementary Figure S8. DLK1 expression was ubiquitous in human ACC and increased the risk of disease recurrence and progression.**

(A) *DLK1* expression is higher in ACC than other adrenal pathologies (*London cohort*, F=5.937, *P* < 0.001). *Post hoc* analyses revealed that the mean H-score in ACC (115.4 ± 89.2) was higher than in each individual group when assessed with multiple comparisons and adjusted *P* values (adrenal adenoma 54.27 ± 56.88, normal adjacent tissue 29.06 ± 39.77, other benign lesions 10.34 ± 19.38, and other malignant lesions 1.806 ± 3.032).

(B-E) The range of *DLK1* expression across the Würzburg cohort is illustrated, showing few positive cells in the tumor parenchyma (B) versus dense, intense staining throughout (C). (D, E) panoramic sections illustrating heterogenous DLK1 expression within individual tumor samples.

(F) *DLK1* expression remained consistent across different ENSAT tumor stages at presentation (ENSAT I&II:130.7 ± 64.97, ENSAT III: 117.4 ± 50.30, ENSAT IV: 121.4 ± 57.67, ANOVA F = 0.731, *P* = 0.483).

(G) No significant difference in *DLK1* expression was observed based on the hormonal activity of tumors (ANOVA F=0.627, *P* = 0.536).

(H) *DLK1* expression was unrelated to Ki-67% (*r* = -0.014, *P* = 0.878)

Higher *DLK1* levels were associated with increased disease recurrence (Figure 1K).

(I-J) This association was more pronounced in ENSAT stage I & II (*n* = 52, median RFS: high *DLK1*, 10 months versus low *DLK1*, 32.5 months; HR = 2.098, 95% CI: 1.127 – 3.903) than in ENSAT stage III & IV (*n* = 36, median RFS: high *DLK1*: 11 months versus low *DLK1*, 18.5 months; HR = 1.648, 95% CI: 0.796 – 3.412).

(K) Higher *DLK1* levels were associated with a trend towards an increased risk of disease progression (*n* = 176, median PFS: high *DLK1*, 7 months versus low DLK1, 8 months; HR: 1.311, 95% CI: 0.954 – 1.801, *P* = 0.080). This trend was also observed when assessing *DLK1* expression in quartiles (log-rank test for trend, χ^2^=2.72). Data are displayed as individual points, with horizontal bars representing the mean.

Abbreviations: ACC, adrenocortical carcinoma; RFS, recurrence-free survival; PFS, progression-free survival, HR, hazard ratio; CI, confidence interval; NAPACA, non-aldosterone producing adrenocortical adenoma. * *P* < 0.05, ** *P* < 0.01, *** *P* < 0.001, **** *P* < 0.0001.


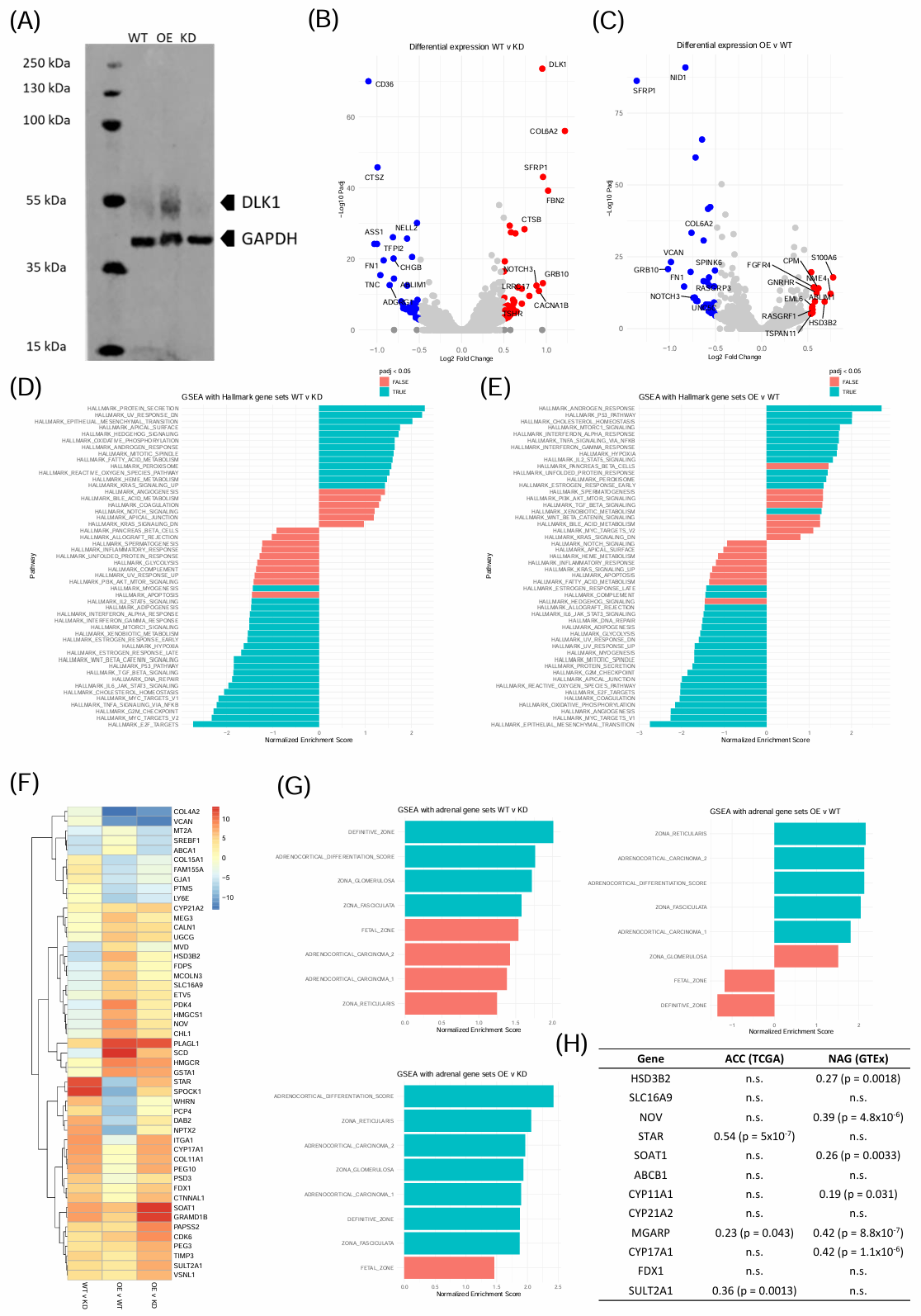


**Supplementary Figure S9.** **Differential gene expression and gene set enrichment analysis in *DLK1*-knockdown and overexpressing cell lines.**

(A) Western blot analysis of DLK1 and GAPDH protein expression in wild-type (WT) H295R cells, DLK1-overexpressing (OE) H295R cells, and DLK1-knockdown (KD) H295R cells.

(B-C) Volcano plots of differentially expressed genes (DEGs) comparing *DLK1*  KD and OE cell lines to WT. The top 10 DEGs (by log2FoldChange) in each direction are labelled, highlighting that some genes, including *NOTCH3* and *GRB10,* are strongly overexpressed in both KD and OE lines.

(D-E) Gene set enrichment analysis (GSEA) using MSigDB hallmark gene sets showing a complex pattern of enrichment of metabolic, proliferative, secretory, and immunomodulatory pathways in KD and OE cell lines, potentially reflecting senescence-associated secretory phenotypes [20, 21]. In particular, proliferation-associated pathways such as G2M checkpoint, E2F, and MYC target genes, as well as immune-related IL6/JAK/STAT3 signaling pathways, are enriched in cells with lower *DLK1* expression. In contrast, epithelial-mesenchymal transition, TP53 response, oxidative phosphorylation (OXPHOS), and cholesterol homeostasis gene sets appear to be associated with DLK1 perturbation.

(F) Heatmap showing expression scores of top DGEs (Wald score statistic > ± 5) within adrenal gene sets. Hierarchical clustering identifies clusters of adrenal genes with similar expression patterns in KD and OE cell lines. A six-gene cluster containing *STAR* exhibits expression patterns similar to *NOTCH3* and *GRB10*, suggesting that *DLK1* perturbation may influence imprinting- or ligand-receptor-based signaling, leading to a complex mixture of adrenal cell phenotypes in KD and OE cultures.

(G) GSEA with adrenal gene sets shows specific enrichment of adrenal definitive zone (DZ) and zona glomerulosa (ZG) gene sets in *DLK1* KD compared to WT, while *DLK1* OE displays enrichment of gene sets associated with zona reticularis (ZR) and malignant ACC cell types. Both KD and OE cell lines show enrichment in the adrenocortical differentiation score (ADS) [12] and zona fasciculata (ZF) gene sets compared to WT, with ADS displaying the strongest enrichment in *DLK1*  OE when compared to KD.

(H) Pearson correlation coefficients for *DLK1* and significant DGEs (adj. *P* < 0.05) from the ADS gene set in bulk ACC and normal adrenal gland (NAG) RNA-seq samples. *STAR* and *SULT2A1* display strong positive correlations with *DLK1* in ACC but not in NAG tissue, while *DLK1* correlates significantly with 6/12 (50%) ADS genes in NAG. Of note, *STAR* expression is negatively correlated with *SULT2A1* in NAG (*r* = -0.3, *P* < 0.001), suggesting that *DLK1* OE in ACC perturbs reciprocal regulation of steroidogenesis pathways.

Abbreviations: WT, wild-type; OE, overexpressing; KD, knockdown; DEG, differential gene expression; GSEA, gene set enrichment analysis; NAG, normal adrenal gland; ACC, adrenocortical carcinoma; ADS, adrenal differentiation score; ZF, zona fasciculata; ZG, zona glomerulosa; ZR, zona reticularis.


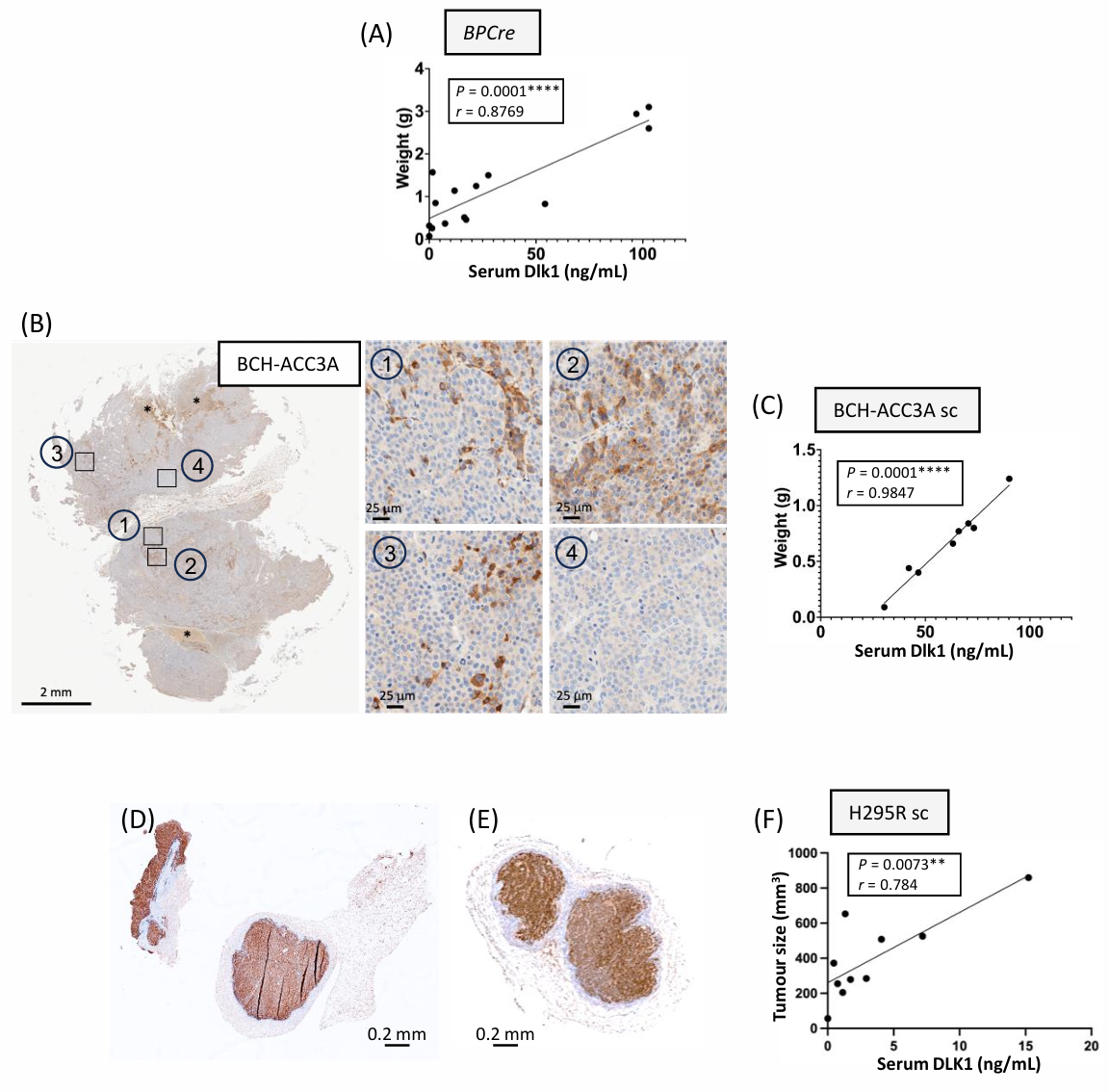


**Supplementary Figure S10. Serum Dlk1 levels were elevated in mouse models of adrenal carcinogenesis.**

(A) Correlation between serum Dlk1 levels and tumor weight in *BPCre* mice.

(B) Immunohistochemical detection of Dlk1 expression in tumors from mice injected subcutaneously with BCH-ACC3A cells, showing varying levels of Dlk1 expression.

(C) Correlation between serum Dlk1 levels and tumor weight in mice injected subcutaneously with BCH-ACC3A cells.

(D-E) Representative images of Dlk1 expression in tumors retrieved from Nu-Nu mice injected with H295R.

Abbreviations:

(F) Correlation between serum Dlk1 levels and tumor size in mice injected subcutaneously with H295R cells.

Abbreviations: sc, subcutaneous. ** *P* < 0.01, **** *P* < 0.0001.

**
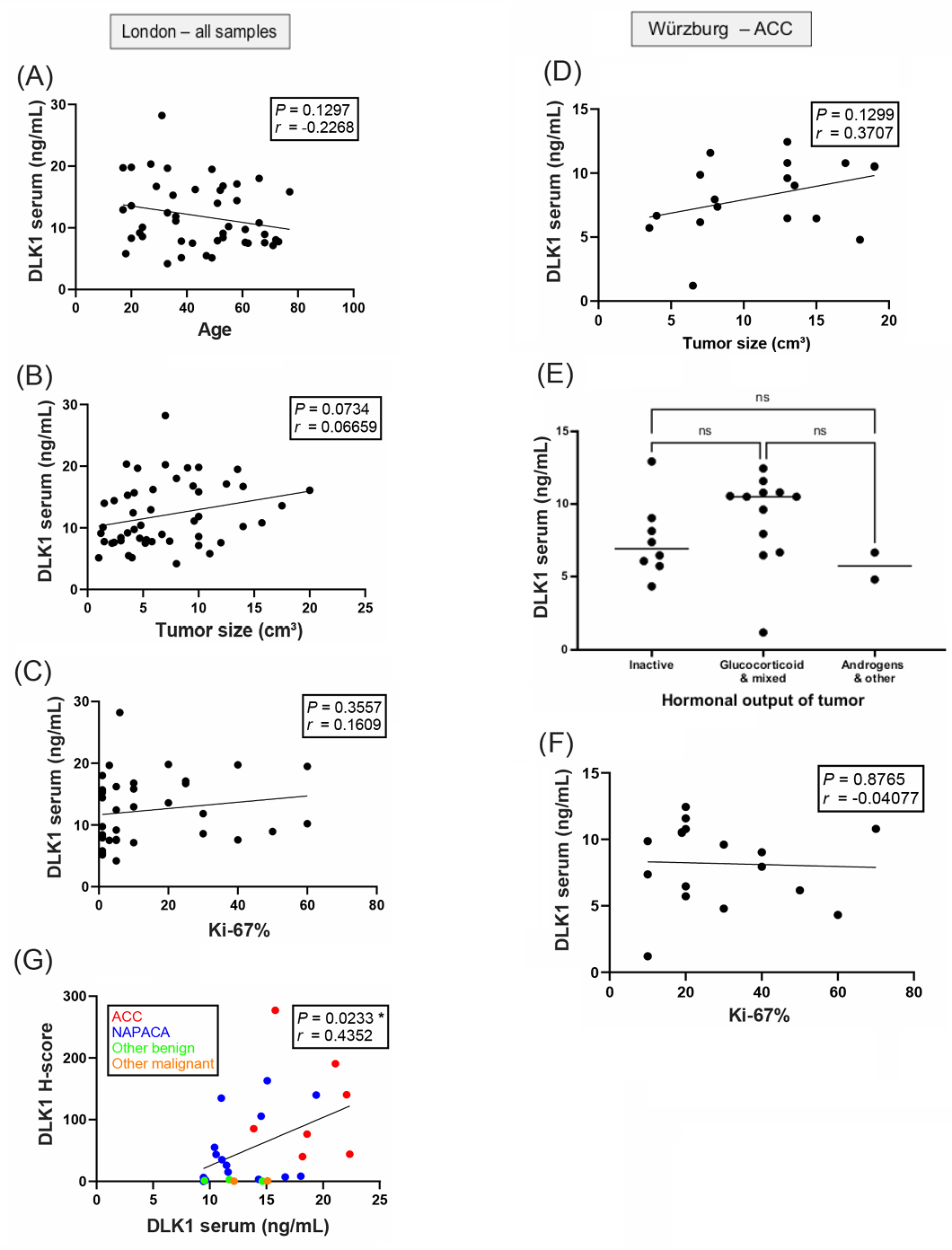
**

**Supplementary Figure S11.** **DLK1 serum levels were not affected by disease presentation**.

(A-C) *London cohort*: DLK1 serum levels showed no significant correlation with age (A), tumor size (B), or Ki-67% (C).

(D-F) *Würzburg cohort*: In ACC alone, DLK1 serum levels showed no significant correlation with tumor size (D), tumor hormonal output (E), or Ki-67% (F).

(G) A significant positive correlation was observed between DLK1 H-score in tissue and serum DLK1 levels in the same patients across the entire London cohort.

Abbreviations: ACC, adrenocortical carcinoma; NAPACA, non-aldosterone producing adrenocortical adenoma.

**
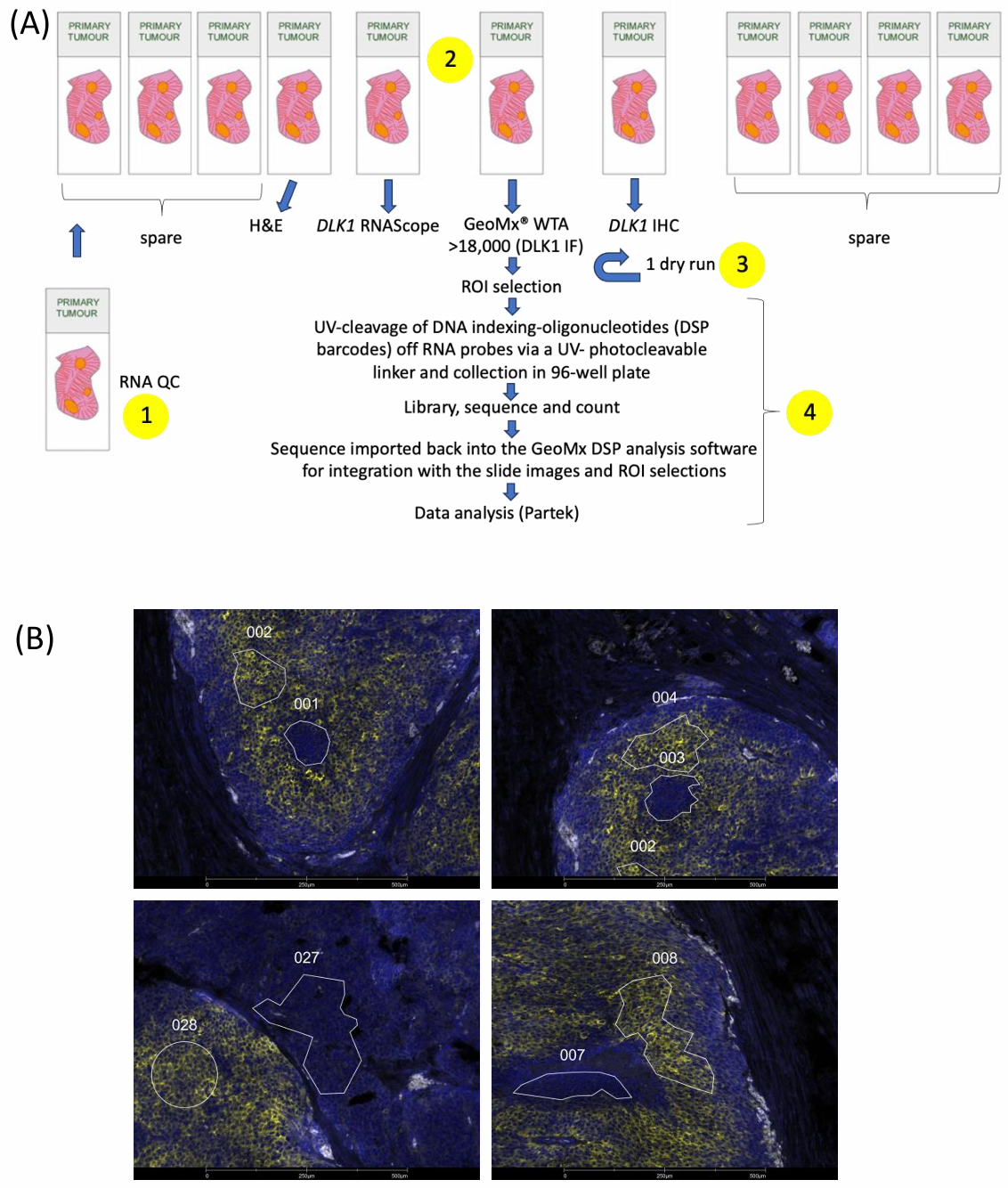
**

**Supplementary Figure S12. Workflow of GeoMx Spatial transcriptomics**.

(A) Consecutive FFPE sections were mounted onto Superfrost Plus slides, and two random sections per ACC were processed for mRNA Quality Control (QC) (step 1). After QC, consecutive sections were processed for Hematoxylin and Eosin (H&E) staining, *DLK1* RNAScope, DLK1 immunohistochemistry (IHC), and DLK1 immunofluorescence (IF) according to the GeoMx protocol (step 2). A dry run was performed for each sample before the experiment to confirm that DLK1 IHC RNAScope signals were identical to those obtained with IF from the GeoMx protocol (step 3). After hybridization with the Whole Transcriptome Atlas (WTA), Regions of Interest (ROI) were selected based on DLK1 signal, and Digital Spatial Profile (DSP) barcodes were collected into 96-well plates (1 ROI/well) via UV-cleavage and microcapillary aspiration, before sequencing the library (Illumina) (step 4).

(B) Examples of ROI from ACCs based on DLK1 expression (ROI 1, 3, 7, 27: DLK1^-^, ROI 2, 4, 8, 28: DLK1^+^). Stroma and vasculature were always DLK1^-^ and excluded.

Abbreviations: H&E, Hematoxylin and Eosin; QC, quality control; IHC, immunohistochemistry; IF, immunofluorescence; ROI, region of interest; DSP, digital spatial profile.

**
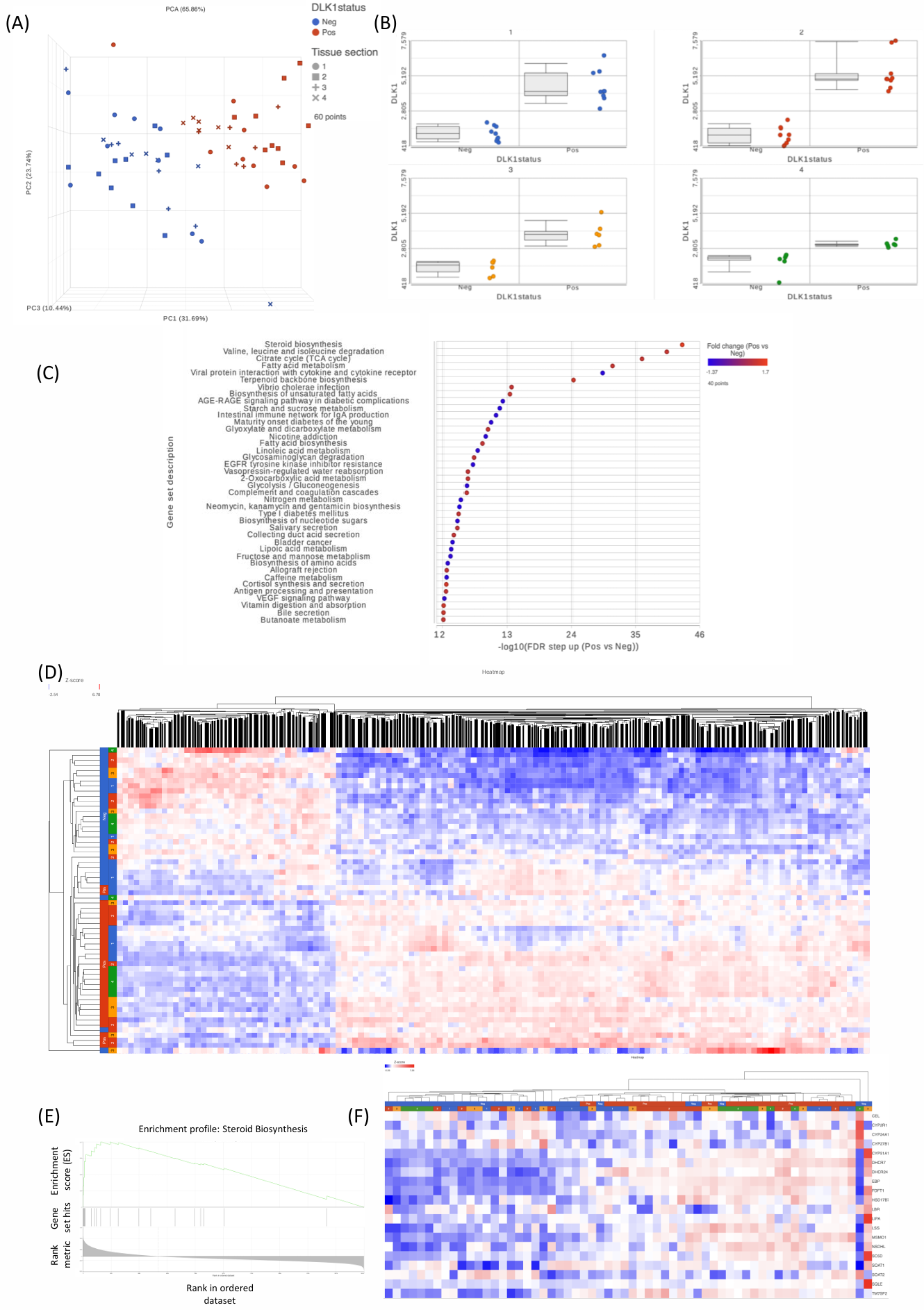
**

**Supplementary Figure S13**. **Spatial transcriptomic data from DLK1^+^ and DLK1^-^ tumor regions.**

(A) Principal component analysis (PCA) plot of the 4 ACC samples analyzed by spatial transcriptomics, showing the clustering of DLK1^+^ and DLK1^-^ tissue areas.

(B) *DLK1* mRNA count in the regions of interest (ROI) from four ACC samples, categorized as either negative (Neg) (expressing low or undetectable levels) or positive (Pos) for DLK1 protein expression.

(C) Gene set ANOVA showing the most differentially regulated pathways in DLK1^+^ and DLK1^-^ tumor areas. The most upregulated pathway was steroid biosynthesis, as shown by gene set enrichment analysis in (E).

(D) Heatmap showing unsupervised clustering of differentially expressed genes between DLK1^+^ and DLK1^-^ tumor areas.

(E) Gene set enrichment profile of the steroid biosynthesis pathway.

(F) Heatmap showing unsupervised clustering of differentially expressed genes in the steroid biosynthesis pathway dataset.

Abbreviations: PC, principal component; Neg, DLK1 negative; Pos, DLK1 positive.

**
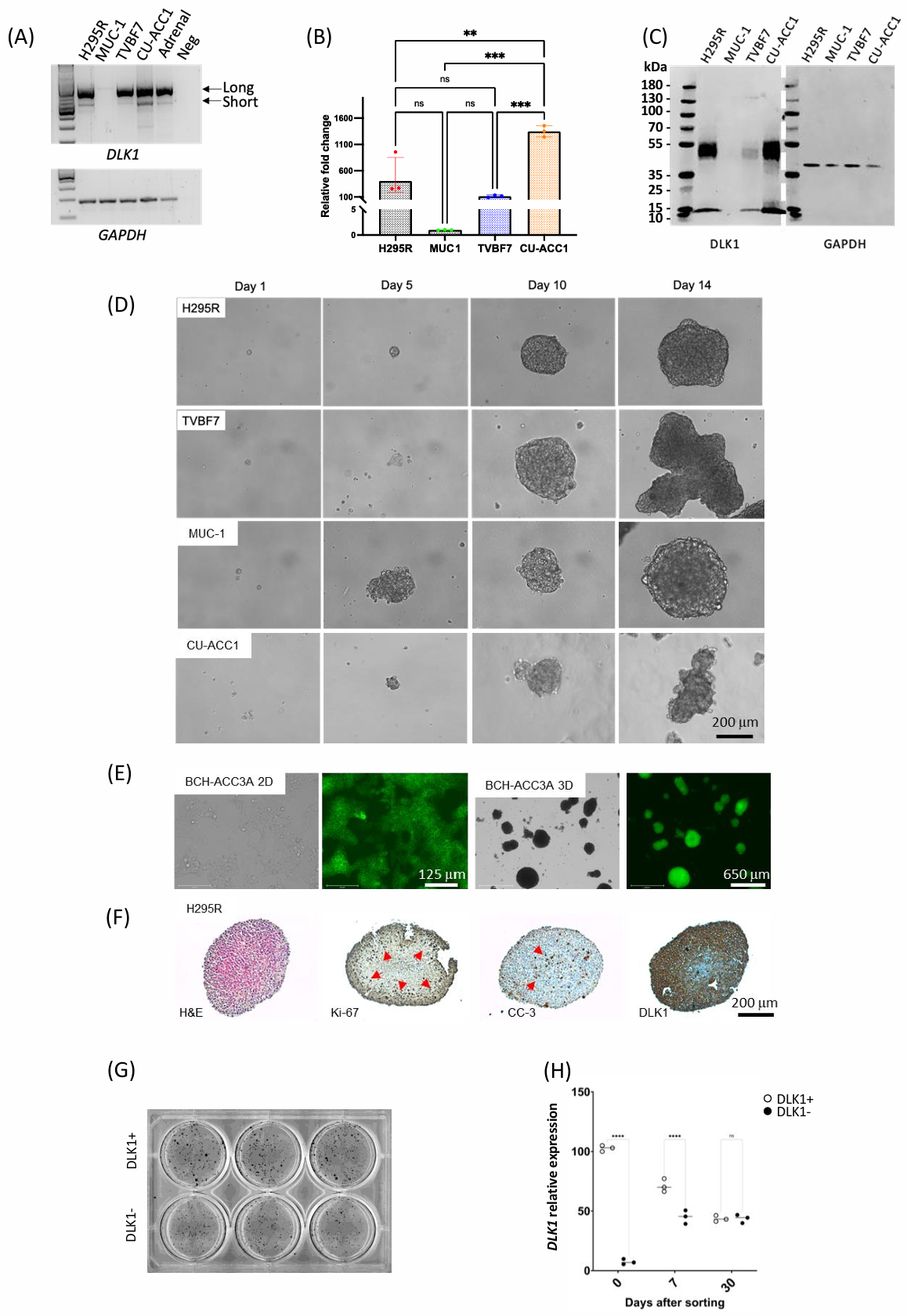
**

**Supplementary Figure S14. DLK1 expression in different ACC cell lines.**

**(**A) PCR analysis of *DLK1* isoform expression in H295R, MUC-1, TVBF7, CU-ACC1, and human adrenal tissue. A preponderance of the full-length *DLK1* isoform was observed in H295R, TVBF7, CU-ACC1, and human adrenal tissue.

(B) TaqMan analysis of *DLK1* mRNA expression in the indicated ACC lines.

(C) Western blot analysis of DLK1 protein expression in the indicated ACC lines.

(D) Time course of spheroids formation in H295R, TVBF7, MUC-1, and CU-ACC1 human ACC cells.

(E) Bright field and fluorescent images of BCH-ACC3A ACC mouse cells grown in adherent (2D) and spheroid (3D) conditions.

(F) Paraffin sections from PFA-fixed H295R spheroids stained with hematoxylin and eosin (H&E), and antibodies against Ki-67, Cleaved caspase 3 (CC-3), and DLK1. The majority of Ki-67^+^ cells are located at the periphery, while signal for CC-3 is mainly in the center of the spheroid, indicating normoxic and hypoxic areas, respectively. Note the strong expression of DLK1.

(G) Representative colony forming units (CFU) from DLK1^+^ and DLK1^-^ fluorescence-activated cell sorting (FACS)-sorted H295R cells.

(H) TaqMan analysis of *DLK1* mRNA expression in DLK1^+^ and DLK1^-^ FACS-sorted H295R cells after sorting, and at 7- and 30-days post-sorting.

Abbreviations: H&E, Hematoxylin and eosin; CC-3, cleaved caspase 3; ns – not significant. ** *P* < 0.01, *** *P* < 0.001, **** *P* < 0.0001.

**Supplementary References**

1. Borges KS, Pignatti E, Leng S, Kariyawasam D, Ruiz-Babot G, Ramalho FS, et al. Wnt/beta-catenin activation cooperates with loss of p53 to cause adrenocortical carcinoma in mice. Oncogene. 2020;39(30):5282-91.

2. Mohan DR, Borges KS, Finco I, LaPensee CR, Rege J, Solon AL, et al. beta-Catenin-Driven Differentiation Is a Tissue-Specific Epigenetic Vulnerability in Adrenal Cancer. Cancer Res. 2023;83(13):2123-41.

3. Bielinska M, Parviainen H, Porter-Tinge SB, Kiiveri S, Genova E, Rahman N, et al. Mouse strain susceptibility to gonadectomy-induced adrenocortical tumor formation correlates with the expression of GATA-4 and luteinizing hormone receptor. Endocrinology. 2003;144(9):4123-33.

4. Kananen K, Markkula M, Mikola M, Rainio EM, McNeilly A, Huhtaniemi I. Gonadectomy permits adrenocortical tumorigenesis in mice transgenic for the mouse inhibin alpha-subunit promoter/simian virus 40 T-antigen fusion gene: evidence for negative autoregulation of the inhibin alpha-subunit gene. Mol Endocrinol. 1996;10(12):1667-77.

5. Bankhead P, Loughrey MB, Fernandez JA, Dombrowski Y, McArt DG, Dunne PD, et al. QuPath: Open source software for digital pathology image analysis. Sci Rep. 2017;7(1):16878.

6. Merritt CR, Ong GT, Church SE, Barker K, Danaher P, Geiss G, et al. Multiplex digital spatial profiling of proteins and RNA in fixed tissue. Nat Biotechnol. 2020;38(5):586-99.

7. Kim D, Langmead B, Salzberg SL. HISAT: a fast spliced aligner with low memory requirements. Nat Methods. 2015;12(4):357-60.

8. Liao Y, Smyth GK, Shi W. featureCounts: an efficient general purpose program for assigning sequence reads to genomic features. Bioinformatics. 2014;30(7):923-30.

9. Love MI, Huber W, Anders S. Moderated estimation of fold change and dispersion for RNA-seq data with DESeq2. Genome Biol. 2014;15(12):550.

10. Subramanian A, Tamayo P, Mootha VK, Mukherjee S, Ebert BL, Gillette MA, et al. Gene set enrichment analysis: a knowledge-based approach for interpreting genome-wide expression profiles. Proc Natl Acad Sci U S A. 2005;102(43):15545-50.

11. Liao Y, Wang J, Jaehnig EJ, Shi Z, Zhang B. WebGestalt 2019: gene set analysis toolkit with revamped UIs and APIs. Nucleic Acids Res. 2019;47(W1):W199-W205.

12. Zheng S, Cherniack AD, Dewal N, Moffitt RA, Danilova L, Murray BA, et al. Comprehensive Pan-Genomic Characterization of Adrenocortical Carcinoma. Cancer Cell. 2016;30(2):363.

13. Iwahashi N, Umakoshi H, Fujita M, Fukumoto T, Ogasawara T, Yokomoto-Umakoshi M, et al. Single-cell and spatial transcriptomics analysis of human adrenal aging. Mol Metab. 2024;84:101954.

14. Tourigny DS, Altieri B, Secener KA, Sbiera S, Schauer MP, Arampatzi P, et al. Cellular landscape of adrenocortical carcinoma at single-nuclei resolution. Mol Cell Endocrinol. 2024;590:112272.

15. Del Valle I, Young MD, Kildisiute G, Ogunbiyi OK, Buonocore F, Simcock IC, et al. An integrated single-cell analysis of human adrenal cortex development. JCI Insight. 2023;8(14):e168177.

16. Tang Z, Li C, Kang B, Gao G, Li C, Zhang Z. GEPIA: a web server for cancer and normal gene expression profiling and interactive analyses. Nucleic Acids Res. 2017;45(W1):W98-W102.

17. Grabek A, Dolfi B, Klein B, Jian-Motamedi F, Chaboissier MC, Schedl A. The Adult Adrenal Cortex Undergoes Rapid Tissue Renewal in a Sex-Specific Manner. Cell Stem Cell. 2019;25(2):290-6 e2.

18. Farahani RM, Xaymardan M. Platelet-Derived Growth Factor Receptor Alpha as a Marker of Mesenchymal Stem Cells in Development and Stem Cell Biology. Stem Cells Int. 2015;2015:362753.

19. Finco I, Lerario AM, Hammer GD. Sonic Hedgehog and WNT Signaling Promote Adrenal Gland Regeneration in Male Mice. Endocrinology. 2018;159(2):579-96.

20. Coppe JP, Patil CK, Rodier F, Sun Y, Munoz DP, Goldstein J, et al. Senescence-associated secretory phenotypes reveal cell-nonautonomous functions of oncogenic RAS and the p53 tumor suppressor. PLoS Biol. 2008;6(12):2853-68.

21. Warde KM, Smith LJ, Liu L, Stubben CJ, Lohman BK, Willett PW, et al. Senescence-induced immune remodeling facilitates metastatic adrenal cancer in a sex-dimorphic manner. Nat Aging. 2023;3(7):846-65.
